# Childhood asthma and physical activity: a systematic review with meta-analysis and Graphic Appraisal Tool for Epidemiology assessment

**DOI:** 10.1186/s12887-016-0571-4

**Published:** 2016-04-18

**Authors:** Lene Lochte, Kim G. Nielsen, Poul Erik Petersen, Thomas A. E. Platts-Mills

**Affiliations:** Department of Odontology, University of Copenhagen, Copenhagen, 1014 Denmark; Department of Pediatrics and Adolescent Medicine, Copenhagen University Hospital, Rigshospitalet, Copenhagen, 2100 Denmark; Department of Medicine, Division of Allergy and Clinical Immunology, University of Virginia, Charlottesville, 22908 VA USA

**Keywords:** Systematic review, Pediatric, Asthmatic disease, Exercise

## Abstract

**Background:**

Childhood asthma is a global problem affecting the respiratory health of children. Physical activity (PA) plays a role in the relationship between asthma and respiratory health. We hypothesized that a low level of PA would be associated with asthma in children and adolescents. The objectives of our study were to (1) summarize the evidence available on associations between PA and asthma prevalence in children and adolescents and (2) assess the role of PA in new-onset or incident asthma among children and adolescents.

**Methods:**

We searched Medline, the Cochrane Library, and Embase and extracted data from original articles that met the inclusion criteria. Summary odds ratios (ORs) and confidence intervals (CIs) were used to express the results of the meta-analysis (forest plot). We explored heterogeneity using funnel plots and the Graphic Appraisal Tool for Epidemiology (GATE).

**Results:**

We retrieved 1,571 titles and selected 11 articles describing three cohort and eight cross-sectional studies for inclusion. A meta-analysis of the cohort studies revealed a risk of new-onset asthma in children with low PA (OR [95 % CI] 1.32 [0.95; 1.84] [random effects] and 1.35 [1.13; 1.62] [fixed effects]). Three cross-sectional studies identified significant positive associations between childhood asthma or asthma symptoms and low PA.

**Conclusions:**

Children and adolescents with low PA levels had an increased risk of new-onset asthma, and some had a higher risk of current asthma/or wheezing; however, there was some heterogeneity among the studies. This review reveals a critical need for future longitudinal assessments of low PA, its mechanisms, and its implications for incident asthma in children. The systematic review was prospectively registered at PROSPERO (registration number: CRD42014013761; available at: http://www.crd.york.ac.uk/PROSPERO [accessed: 24 March 2016]).

**Electronic supplementary material:**

The online version of this article (doi:10.1186/s12887-016-0571-4) contains supplementary material, which is available to authorized users.

## Background

Asthma is one of the most common chronic pediatric diseases [1]. The prevalence of asthma in children has increased over the last thirty years in most developed countries [2, 3], although the prevalence has started to decrease in adolescents in Western countries [4, 5]. The etiology of childhood asthma is still not understood [6, 7], and the increase in prevalence has not been fully explained [8]. Physical activity (PA) is known to be associated with asthma symptoms in asthmatic children [9, 10], but its role in asthma prevention is unclear.

In Europe, PA levels have declined in children and adolescents [11]. Physical conditioning programs may reduce childhood asthma symptoms [12–14]; moreover, studies of asthmatic children have indicated that PA may induce anti-inflammatory effects [15, 16] such that brief intervals of PA alter the immune response [15]. However, whether such effects [17, 18] translate into a reduced risk of developing asthma also remains unclear.

The decline in PA may be linked to the increased prevalence and severity of childhood asthma [7, 9, 19, 20] or even to undiagnosed asthma [21]. Cross-sectional studies have shown inconsistent associations between PA and childhood asthma. In some studies, low levels of PA were related to a high asthma risk [22–24]; however, other studies did not find an association [25]. The few longitudinal studies on PA and childhood asthma have produced diverse results; in fact, one study showed that high levels of PA were related to an increase in diagnosed asthma [26].

Few authors [27] have collated the results of observational studies in this field. Therefore, our objectives were to (1) summarize the available evidence on associations between PA and asthma prevalence in children and adolescents and (2) assess the role of PA in new-onset or incident asthma in children and adolescents. We report the hypothesized associations between low PA and asthma in children and adolescents.

## Methods

### Design

This study was a systematic literature review that included a quantitative analysis (meta-analysis) and assessments using the Graphic Appraisal Tool for Epidemiology (GATE) [28]. We identified published studies examining the associations between PA and asthma in children and adolescents.

The protocol followed the Centre for Reviews and Dissemination (CRD) guidelines [29] for conducting systematic reviews: we (1) identified the available research and selected studies for inclusion, (2) extracted data, (3) assessed and described study quality, and (4) synthesized our findings. The reporting of our findings adhered to the Preferred Reporting Items for Systematic reviews and Meta-Analyses (PRISMA) statement [30] and, initially, to the consensus statement of the Meta-analysis Of Observational Studies in Epidemiology (MOOSE) Group [31]. Additional file 1 presents the PRISMA [32] checklist items that we examined. Additional file 2 presents the details obtained from using the Reporting Checklist of the MOOSE Group [31]. We used the GATE approach [28] to illustrate and assess the quality of the studies that did not qualify for the meta-analysis. When possible, we summarized the individual quality of these studies, assessing errors, effect sizes, and study applicability. For the meta-analysis, we used data on exposure to PA provided for asthma and control children; the outcomes were new-onset childhood asthma/or wheezing.

### Ethical aspects

Since this is a systematic review based on published literature, the ethical requirements have been met previously for each individual study. Accordingly, the relevant approvals are stated in each original publication (article) included in our review. Written informed consent was obtained from the patient's guardian/parent/next of kin for the publication of each original article included in this report and any accompanying images.

### Inclusion criteria for studies on PA and asthma diagnoses

We included longitudinal and cross-sectional studies that investigated asthma and PA in children and adolescents aged 0–18 years. PA was documented by either interviews or self-administered questionnaires. Childhood asthma was defined using parental reports of either physician diagnosis of asthma, “current” (within last 12 months) asthma, “ever” (lifetime) asthma, wheezing, exercise-induced asthma (EIA), or medical treatment of asthma symptoms. We defined new-onset asthma (incident asthma) as a physician diagnosis of asthma/or wheezing. Hence, for incident asthma, there was no sampling based on disease status [33]. We used asthma/or wheezing (a representative asthma symptom) [34] to capture the heterogeneous symptomatology of asthma in children [35].

We defined PA as a behavioral concept that varied according to “leisure time” or “sports and exercise” [36]. We recognized that PA can be further characterized by its dimensions as follows: (1) frequency, (2) intensity, (3) duration, and (4) type [37]. Intensity has been identified as the key dimension for possible dose-response relationships with either reduced or increased health risks for exercise-induced medical conditions [38]. This review did not distinguish between PA and exercise. The concept “PA” referred to general leisure-time PA, exercise, or sports during or outside of school hours [39]. High amounts of TV viewing (duration in hours) represented sedentary behavior [40, 41] and were used as a proxy for low PA. This approach was based on the previous use of TV viewing [24, 42] which validated that TV viewing could be used to represent PA in population surveys. It was beyond the scope of this review to discuss the scientific distinctions between sedentary activity and physical inactivity in children and adolescents.

### Inclusion criteria for the meta-analysis

We adhered to appropriate standards [29] in defining our criteria for the meta-analysis, which were as follows: (1) broadly similar research questions, (2) comparable participant populations (children and adolescents), and (3) broadly similar research mechanisms.

### Exclusion criteria

**Fig. 1:**
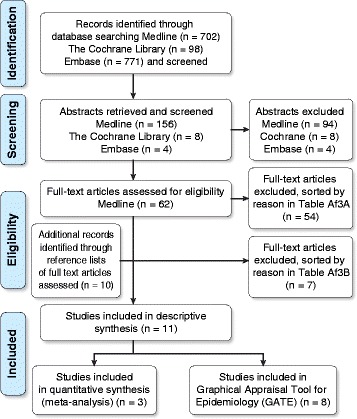
Inclusion and Exclusion Criteria for Systematic Reviews. Numbers of search results from Medline, the Cochrane Library, and Embase

We excluded studies involving adults >18 years of age and non-English-language studies [43]. We also excluded single outcomes of intermediate phenotypes for childhood asthma (i.e., bronchial hyperresponsiveness [BHR], allergic rhinoconjunctivitis, atopic dermatitis, airway inflammation, eczema) and cumulative incidence along with studies that had fitness or body composition as their only outcomes. Studies that reported on only PA or asthma were excluded, as were clinical investigations (e.g., randomized controlled trial [RCT] designs) of training and/or medical treatment in children with asthma. If pediatric asthma or PA was explored using noncomparable (rare) methodologies or the studies excluded relevant participants, the studies were excluded. We excluded other reviews, methodology reports, validation studies, and studies that collected data for other purposes or had other non-applicable outcomes. The two stages of exclusion are illustrated in Fig. 1, and the articles excluded at each stage are grouped by exclusion rationale in Additional file 3A and B.

### Search strategy

#### Identifying studies and study selection

We searched the following databases: Medline, National Library of Medicine (1946 to the last search date: 7 Jan 2014), the Cochrane Library (all Cochrane products to the last search date: 13 Jan 2014), and Embase/Excerpta Medica (2013 to the last search date: 17 Jan 2014). We used medical subject headings (MeSH) for asthma/or wheezing and PA. In Medline, “physical activity” was not available as a MeSH heading, and therefore we included the MeSH headings “physical fitness”, “exercise”, and “physical exertion”; we also restricted the search to English language, humans, and age 0–18 years. Table 1 illustrates the full electronic search strategy used in Medline. Initially, to expand the search, we conducted exploratory text, title, and adjacent word searches. Because we obtained large numbers of unrelated titles, these searches were subsequently omitted. One medical subject librarian (CFB) reviewed our search strategies for the Cochrane and Medline databases to ensure that the variation in search terms across the databases was taken into account. We read review articles and identified additional studies from the reference lists of retrieved full-text articles.

**Table 1:** Full Electronic Search Strategy for Medline

Action	Term
1	^a^asthma
2	^a^bronchial hyperreactivity
3	^a^bronchoconstriction
4	^a^respiratory hypersensitivity
5	^a^respiratory sounds
6	^a^dyspnea
7	^a^asthma, exercise-induced
8	^a^respiratory function tests
9	^a^exercise test
10	1 or 2 or 3 or 4 or 5 or 6 or 7 or 8 or 9
11	^a^physical fitness
12	^a^exercise
13	^a^physical exertion
14	11 or 12 or 13
15	10 and 14
16	Limit 15 to English language
17	Limit 16 to humans
18	Limit 17 to "all child (0 to 18 years)"

^a^Indicates a focused search using medical subject heading (MeSH) terms. “Or” was used to combine related search terms. “And” was used to combine two sets of terms for asthma and physical activity

LL searched and screened studies by title and abstract for eligibility. Two medical students declined to be independent reviewers, and LL identified the articles for inclusion. When necessary, assessment was performed by the lead investigator (PEP). Figure 1 presents a flow diagram illustrating the studies identified by the database searches.

### Data extraction and study quality

LL extracted information from the included studies. Table 2 shows the information points that were extracted from each study for the descriptive data synthesis. The extracted items represented adopted standards for methods, participants, outcomes, and results as defined in the checklist of The Cochrane Handbook for Systematic Review [44]. For the quantitative data synthesis (the meta-analysis), we extracted individual summary data [29] from each study that met the criteria for meta-analysis. We excluded BHR as an asthma phenotype and consequently were only able to obtain asthma severity data from a few of the reviewed studies [22, 24, 45].

**Table 2:** Data Extracted from Individual Studies in the Systematic Review

Data	
Name of first author	
Year of publication	
Study design	
Age (years) of study population: Mean (±2 SD) or range	
Definition of physical activity	
Definition of asthma	
Number of children with asthma and total study population size	
Main effect size and confidence interval	
Adjustment covariates	
Key conclusions of the study authors	

Using GATE [28] entailed documenting the study population, representativeness, measurement(s), and timing. All data that were extracted to electronic GATE forms [46] are illustrated in Additional file 4.

### Statistical methods

The studies we examined followed different protocols, and therefore, we explored the clinical and methodological sources of their heterogeneity by reviewing the descriptive study characteristics that we extracted (Table 2). For the meta-analysis, we reported both random- and fixed-effects models (using inverse variance [29]) to illustrate the respective inter- and intra-study variability [47]. Technically, we produced 2 × 2 tables; i.e., we entered the numbers of children who developed asthma in the exposed (low PA) and unexposed (high PA) groups [48]. This approach produced summary statistics for each individual study and an overall estimate, both of which were expressed as odds ratios (ORs) and 95 % confidence intervals (CIs). Forest plots were used to illustrate these summary statistics and the variation (heterogeneity) across the studies. We expressed the percentages of variability in the effect estimates that were attributable to between-study variation (heterogeneity) rather than chance using I-squared (I^2^), and the statistical assessment was performed using the chi-squared (χ^2^) test [29, 47]. We assessed the risk of publication bias or selective outcome reporting [30] across studies by estimating the standard errors (SEs) of the logarithmic (log) scale ORs (logORs), and we depicted these graphically on the horizontal (logORs) and vertical (SEs) axes of a funnel plot. In addition, we assessed the funnel plot for asymmetry [49]. We used STATA™ version 12 (StataCorp, College Station, TX, US) [50] for the calculations and *P* set at 5 %.

## Results

### Identified studies

The searches yielded a total of 1,571 titles, and 11 studies that examined PA and childhood asthma met the inclusion criteria. Initially, we removed duplicates and contacted the authors of two articles to clarify details regarding the original data. Both authors responded, and we obtained the full texts of 62 studies. Of the 11 studies that met the inclusion criteria, three were cohort studies [45, 51, 52], and eight were cross-sectional studies [9, 22–25, 53–55]. We excluded 54 studies followed by seven additional studies at two different stages (Fig. 1). Tables 3 and 4 present the data extracted from each study sorted by study design. Below follow reports on the cohort studies (including meta-analysis) and the cross-sectional studies given in separate sections.

**Table 3:** Cohort or Longitudinal Studies Included in the Systematic Review by Selected Study Information

Author	Year	Study design	Age^a^ (years)	Definition		Number		Main effect size Adj OR/HR/GMR/mean	Adj covariates	Key conclusions reported by the study authors
				*Physical activity*	*Asthma*	*Asthma*	*Total*			
Vogelberg	2007	Cohort follow-up 6–7 years	16–18	Sports freq(questionnaire)	Physician diagnosed (wz)	329^wz,b^	2,858^b^	Risk (OR [95 % CI]) of incident wz by sports >3 times per wk vs ≤ once per month (rfgr): 0.8 (0.5–1.3)	Active and passive smoking, BMI, SES, gender	Inverse associations between wz and sport or PC
Sherriff	2009	Cohort follow-up 11.5 years	11.5	TV viewing (questionnaire)	Physician diagnosed	78^b^	1,599^b^	Associations (OR [95 % CI]) of asthma at age 11.5 years with TV viewing at age 3.5 years (>2 hrs/day) vs 1–2 hrs/day (rfgr): 1.8 (1.2–2.6) (*P* trend = 0.0003)	BMI, maternal asthma/allergies and smoking, social variables	Longer duration of TV viewing associated with development of asthma in later childhood
Islam	2009	Cohort follow-up 10 years	7–11+	Team sports (questionnaire)	Physician diagnosed	142^b^	1,580^b^	Associations (HR [95 % CI]) of GSTP1^c^ genotypes with new-onset asthma by > two team sports vs none (rfgr): 2.66 (1.2–5.9) (*P* < 0.05) ^c^Subclass of GST	Ethnicity, community of residence, genetic information (GSTM1^c^ and SNP1/SNP3) ^c^Subclass of GST	Children with Val^105^ variant allele may be protected against increased risk of asthma by exercise

*Adj* Adjusted or adjustment, *BMI* Body mass index, *CI* Confidence interval, *Freq* Frequency, *GST* Glutathione S-transferase, *Hr/hrs* Hour/hours, *HR* Hazard ratio, *OR* Odds ratio, *Rfgr* Reference group, *SD* Standard deviation, *SES* Socio-economic status, *SNP* Single nucleotide polymorphism, *Vs* Versus, *Wk* Week, *Wz* Wheezing

^a^Age: Mean (±2 SD) or range

^b^Those who contributed data on asthma/wheezing and physical activity to the meta-analysis

^c^Subclass of GST

**Table 4:** Cross-Sectional Studies Included in the Systematic Review by Selected Study Information

Author	Year	Study design	Age* (years)		Definition		Number		Main effect size Adj OR	Adj covariates	Key conclusions reported by the study authors
					*Physical activity*	*Asthma*	*Asthma*	*Total*			
Nystad	1997	Cross-sectional	7–16 Area I–III		HBSC (WHO), two questions (hrs/wk and freq/wk)	ISAAC questionnaire and question on current asthma from reference	222 Area I: 123 II: 69 III: 30	4,021 Area I: 2,188 II: 1,045 III: 788	Association (OR [95%CI]) between current asthma and PA 1–3 hrs/wk vs ≤0.5 hr/wk (rfgr): 1.0 (0.6–1.5)	Age, gender, study area	Asthmatic children as physically active as peers
Nystad	2001	Cross-sectional	7–16		HBSC (WHO), two questions (hrs/wk and freq/wk); only hrs reported in article	ISAAC questionnaire plus question about current asthma (from ATS-MRC)	116^wz^	2,112	Associations (OR [95%CI]) wz or whistling (all children) and PA ≤1 hr/wk: 1.9 (0.9–3.8) 2–3 hrs/wk: 2.6 (1.3–5.2) ≥4 hrs/wk: 2.5 (1.2–4.9) vs none (rfgr) “No clear dose-response relationship, but the effect was mainly among active vs inactive children”	Age, atopy (eczema and/or hay fever), current asthma, gender	Positive associations between PA and wz
Lang	2004	Cross-sectional	6–12		Questionnaire 1 ) total mins active in one (1) day; 2) number of days active in typical wk	Questionnaire. Medical provider ever-diagnosed asthma and some asthma symptoms in last 12 months	137	243	Association (OR [95%CI]) between mod/severe persistent asthma and PA <30 mins/day (inactivity) vs all other PA-groups (rfgr): 3.00 (1.19–7.52) (*P*<0.05)	Gender, health beliefs (e.g., child can do as much PA as children similar age without asthma or child upset with strenuous activity)	Disease severity and parental health beliefs contributed to lower activity levels of children with asthma
Jones	2006	Cross-sectional	9–12th grade		PA-levels (questionnaire)	Questionnaire. Physician-diagnosed asthma denoted lifetime asthma with/ without current asthma last 12 months	1,943	13,553	Association (OR [95%CI]) between asthma status and sufficient mod PA: 1.1 (0.9–1.3)	Grade, race/ethnicity, gender	No differences in participation in vig or mod PA among students with and without current asthma
Priftis	2007	Cross-sectional	10–12	PA questionnaire (PANACEA)		ISAAC questionnaire. Asthma symptoms, e.g., ever asthma or ever wz	166^Symptoms^	700	Associations (OR [95%CI]) for asthma symptoms in boys; girls not participating in any PA vs no participation last wk (rfgr): 2.17 (1.34–3.54) (*P*<0.05); 1.63 (0.86–3.11)	Body weight (per 5 kg), time of watching TV or playing video games per day (per 1 hr)	PA associated with reduced odds of reporting asthma symptoms
Corbo	2008	Cross-sectional	6–7	PA levels in regular sports (i.e., formal games or other aerobic exercise) (questionnaire)		ISAAC questionnaire. Defined current asthma	1,343	20,016	Association (OR [95%CI]) between current asthma and low freq of regular sports (1–2 times per wk) vs none (rfgr): 1.13 (0.93–1.38) (*P* trend = 0.069)	Age, BMI, dietary variables, family asthma or rhinitis, mold, parental education and smoking, person filling questionnaire, regular sports, season, gender, study center, TV viewing	Wz or asthma not associated with regular sports activity
Kosti	2012	Cross-sectional	10–12	PA questionnaire (PANACEA)		ISAAC questionnaire. Asthma symptoms, e.g., ever asthma or ever wz	228	1,125	Association (OR [95%CI]) between leisure-time PA and asthma symptoms: 0.90 (0.79–1.03) (Ns)	Age, BMI, KIDMORE score, gender, urban/rural	Inverse relationship between asthma symptoms and leisure PA (rural)
Mitchell	2013	Cross-sectional	6–7 and 13–14	Weekly vig PA (freq) (questionnaire)		ISAAC questionnaire, i.e., ever asthma	Data not given	76,164 (6–7 years) 201,370 (13–14 years)	Associations (OR [95%CI]) between reported asthma ever and PA once or twice per wk vs vig PA never or occasionally each wk (rfgr): 0.96 (0.89–1.04) (6–7 years) 1.14 (1.08–1.20) (13–14 years)	BMI, income, language, region, gender, TV viewing	Vig PA positively associated with symptoms of asthma in adolescents but not in children

*Adj* Adjusted or adjustment, *Age: Range, *ATS-MRC* American Thoracic Society and Medical Research Council, *BMI* Body mass index, *CI* Confidence interval, *Freq* Frequency, *Hr/hrs* Hour/hours, *HBSC* Health Behaviour in School-aged Children, *ISAAC* International Study of Asthma and Allergies in Childhood (ever asthma and wz last 12 months) [2], *KIDMORE index* Mediterranean Diet Quality Index for children and adolescents (total scores and categories described in article), *Min/s* Minute/s, *Mod* Moderate, *Ns* Non-significant, *OR* Odds ratio, *PA* Physical activity, *PANACEA* The Physical Activity, Nutrition and Allergies in Children Examined in Athens Study, *Rfgr* Reference group, *Vig* Vigorous, *Vs* Versus, *Wk* Week, *Wz* Wheezing

### Cohort studies

#### Measurements of new-onset (incident) asthma/or wheezing

Two studies [45, 51] described cases of new-onset asthma using a physician’s diagnosis of asthma (Table 3), and one study described new-onset wheezing [52]. We synthesized three cohort studies [45, 51, 52] that met the criteria for inclusion in our meta-analysis. The follow-up times (in years) were 6–7 [52], 10 [51], and 11.5 [45] (Table 3). In these studies [45, 51, 52], a total of 549 children had new-onset asthma/or wheezing, and the total number of cohort children studied was 6,037 (Table 3). The reported asthma prevalence was 6.0 % [45], the new-onset wheezing prevalence was 11.3 % [52], and the asthma incidence rate was 16.6 % per 1,000 person-years [51]. Overall, 57.7 % (317) of the cohort children with new-onset asthma/or wheezing had low PA [45, 51, 52] (Table 5).

**Table 5:** Distribution (N, %) of Children with New-Onset Asthma/or Wheezing and All Children According to PA

First author, publication year	PA-exposure levels	New-onset asthma outcome	New-onset asthma, N (%)	All children N (%)
Vogelberg et al., 2007 [52]	*Low PA if sport freq ≤ once/wk* High PA (rfgr) if sport freq ≥ two times/wk	Wheezing	*Low PA: 199 (60.5)* High PA: 130 (39.5)	*Low PA: 1,470 (51.4)* High PA: 1,388 (48.6)
Sherriff et al., 2009 [45]	*Low PA if TV viewing* * ≥1 hr/day* High PA (rfgr) if TV viewing = none or <1 hr/day	Asthma	*Low PA: 61 (78.2)* High PA: 17 (21.8)	*Low PA: 1,100 (68.8)* High PA: 499 (31.2)
Islam et al., 2009 [51]	*Low PA if number of team sports = none* High PA (rfgr) if number of team sports ≥1	Asthma	*Low PA: 57 (40.1)* High PA: 85 (59.9)	*Low PA: 648 (41.0)* High PA: 932 (59.0)

Details regarding data from the meta-analyzed studies

*Freq* Frequency, *Hr/hrs* Hour/hours, *N* Number, *PA* Physical activity, *Rfgr* Reference group, *Wk* Week

### Results from meta-analysis

**Fig. 2:**
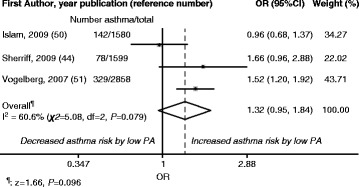
Random-Effects Model: Study-Specific and Overall Odds Ratios (ORs) with 95 % Confidence Intervals (CIs). Data are derived from the meta-analysis of low physical activity (PA) and new-onset asthma during childhood. High PA: Reference category

**Fig. 3:**
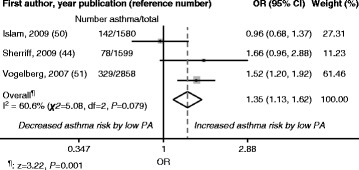
Fixed-Effects Model: Study-Specific and Overall Odds Ratios (ORs) with 95 % Confidence Intervals (CIs). Data are from the meta-analysis of low physical activity (PA) and new-onset asthma during childhood. High PA: Reference category

We conducted a meta-analysis using data on asthma and PA provided by three articles [45, 51, 52]. To combine the study results, we reclassified the exposure variables. The original PA variables were number of team sports played (none, 1–2, >2) [51], sports participation frequency (≤once per month, ≤once per week, 2–3 times per week, >3 times per week) [52], and duration of TV viewing (not at all, <1 hour per day, 1–2 hours per day, >2 hours per day) [45]; for the meta-analysis, we dichotomized the results into no team sports played (low PA) and ≥1 team sport played (high PA) [51], sports participation ≤ once per week (low PA) and ≥2 times per week (high PA) [52], and TV viewing ≥1 hour per day (low PA) and <1 hour per day (high PA) [45]. The reference category was high PA in both the random- and fixed-effects models. The overall meta-analysis results showed positive risks for new-onset asthma (OR [95 % CI] 1.32 [0.95; 1.84] [random effects] and 1.35 [1.13; 1.62] [fixed effects]) in children with low PA compared with high PA (reference). These results are illustrated in Fig. 2 (random effects) and Fig. 3 (fixed effects). I^2^ was 60.6 % (χ^2^ = 5.08, *P* = 0.079) for both random and fixed effects.

### Consistency of meta-analysis results: risk of bias across studies

**Fig. 4:**
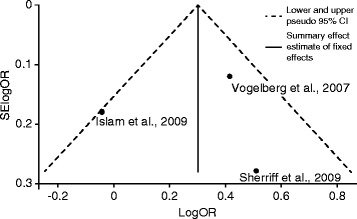
Funnel Plot with 95 % Pseudo Confidence Intervals (CIs). Data are from the meta-analysis depicting the log-scale odds ratios (logORs) (horizontal axis) for new-onset childhood asthma by low physical activity (PA) using individual study effect size data plotted against the standard errors (SEs) (vertical axis) of the logORs

In Fig. 4, the studies that included larger numbers of asthmatic participants [51, 52] were positioned toward the top, i.e., the upper two-thirds of the funnel, representing large sample sizes and small standard errors. Figure 4 also shows that the studies in the meta-analysis [45, 51, 52] were within the 95 % confidence limits (diagonal, dashed lines) around the summary estimate.

### Validity and quality: risk of bias within studies

Our review showed that these three studies [45, 51, 52] explored the role of the temporal sequence following quantified PA exposure and its effect on new-onset asthma/or wheezing in children and adolescents.

### Cross-sectional studies

#### Measurements of current or ever (prevalent) asthma/or wheezing

As shown in Table 4, two studies [22, 53] defined current asthma using questionnaires and a medical provider or physician diagnosis of asthma, whereas a majority [9, 23–25, 54, 55] used the International Study of Asthma and Allergies in Childhood (ISAAC) definitions.

### Validity and quality: risk of bias within studies

We applied the GATE approach developed for the critical appraisal of quantitative studies (electronic forms) [28, 46]. When data were available, we first extracted study numbers regarding exposure, comparison, and outcomes for the association between PA and childhood asthma (Additional file 4). We first used the GATE calculator (one-page Microsoft Excel format) and then transferred the calculated results to the GATE-lite form (one-page Microsoft Word format) [46]. We used GATE to illustrate individual study designs and study details as recommended for gauging bias risks [56].

We illustrated the study design using the acronym PECOT, i.e., extracted data on **p**articipants, **e**xposure, **c**omparisons, **o**utcomes, and **t**ime. To assess study validity, we used the acronym RAMBOMAN, i.e., extracted data on **r**ecruitment, **a**llocation, **m**aintenance, **b**lind or **o**bjective **m**easurements, and **an**alyses.

We applied the GATE approach to a total of eight non-meta-analyzed cross-sectional studies [9, 22–25, 53–55] that investigated asthma prevalence or asthma symptoms. The studies included a total of 4,155 children with current asthma/or wheezing, and the total number of participants was 41,770 children (Table 4 and Additional file 4). Unfortunately, in one study [23], the absolute number of participants was not given. The prevalence of asthma/or wheezing in six of these studies [9, 24, 25, 53–55] ranged from 3.8 % [55] to 23.7 % [24] (Table 4). Five cross-sectional studies originated from Europe [9, 24, 25, 54, 55], and two were from North America [22, 53]. One study was cross-national [23] and included data for 6–7-year-olds from 17 countries and data for 13–14-year-olds from 35 countries (Additional file 4). A majority of the eligible populations were derived from respective national surveys [9, 24, 25, 53–55] of children and adolescents (Additional file 4).

The GATE assessment showed that all eight studies [9, 22–25, 53–55] included measures of exposure and outcome and included a comparison group, and all authors reported the results of adjusted analyses; however, for six studies [22–25, 53, 54], we were unable to obtain data on either the exposure or the comparison groups (Additional file 4). The response rates were >50 % in seven [9, 23–25, 53–55] of the eight studies, although Mitchell et al. [23] observed a response rate <50 % for younger children (6–7 years of age) (Additional file 4). Two cross-sectional studies [22, 53] analyzed PA as an outcome.

The definitions of PA varied. Nystad [55] and Nystad et al. [9] measured PA outside of school hours (sports or exercise) that caused a child to become sweaty or out of breath. Lang et al. [22] registered the total minutes spent engaging in PA in one day, Jones at al. [53] assessed sufficient moderate PA (e.g., fast walking, slow bicycling), and Priftis et al. [24] examined sports-related PA (e.g., brisk walking, running, swimming). Corbo et al. [25] registered PA as regular sports, i.e., formal games or forms of aerobic exercise. Kosti et al. [54] observed leisure-time PA, i.e., unstructured outdoor PA involving play, walking, or cycling. Mitchell et al. [23] described PA as weekly vigorous activity that was sufficient to cause heavy breathing in the child.

Additionally, the definition of low PA varied, with some studies defining low PA as ≤1 hour per week [9], <30 min per day [22], once or twice per week [23], no participation in any PA [24], sports 1–2 times per week [25], sufficient moderate PA [53], leisure-time PA [54], and 1–3 h per week [55]. In four [9, 24, 25, 55], one [23], and one [54] of the eight [9, 22–25, 53–55] cross-sectional studies, the reference groups in the adjusted analyses were “low to no PA”, “no vigorous PA”, or “no leisure time”, respectively (Additional file 4).

In four of the eight studies [9, 22, 53, 55], we were able to extract data for the GATE calculator to estimate occurrences in exposure groups and/or exposure effects. In the studies that investigated distinct low PA (≤1 h per week [9], 1–3 h per week [55], <30 min per day [22]), the occurrences per 100 persons in the exposure groups (EGO) were 6.2 [9], 4.1 [55], and 14.6 [22]. Two [9, 55] of these studies provided sufficient data to estimate exposure effects in terms of relative risk (RR) (Additional file 4). In the remaining four studies [23–25, 54], we could not derive appropriate data for the calculations.

Although we found each of the eight cross-sectional studies [9, 22–25, 53–55] applicable to practice (Additional file 4), the GATE analysis illustrated variations across the studies. We concluded that the quality of these studies was high for cross-sectional designs, but the variation among the studies confirmed that individual study analysis (e.g., GATE assessment) as opposed to common estimation across studies (e.g., meta-analysis) was a sound approach that agreed with recommendations [29, 49, 57].

### All studies - measurements PA

In all but one study, PA was assessed using a questionnaire that asked about sports participation (Tables 3 and 4). One cohort study [45] reported TV viewing (Table 3).

### Main effect size and adjustment covariates

Tables 3 and 4 show that six studies reported positive associations between asthma/or wheezing and low PA [9, 22, 24, 25, 45, 53], and one study showed that asthma/or wheezing was positively associated with high PA [51]. Of the eight cross-sectional studies [9, 22–25, 53–55], three [22–24] indicated significant positive associations between childhood asthma or asthma symptoms and low PA (of which one [23] reported this association for 13- to 14-year-olds only and one [24] reported this association for boys only).

The adjustment covariates applied in the multivariate analyses varied across the reviewed studies. All authors adjusted for age, gender, weight, and/or smoking measures (Tables 3 and 4), three studies included adjustments for asthma history [9, 25, 45], and eight studies adjusted for socioeconomic measures [23, 25, 45, 51–55].

### Summarizing meta-analysis and GATE review

In each section, we first reported the descriptive data syntheses and then the analytical data syntheses based on quantitative (meta-analysis) and qualitative (GATE) approach. Meta-analysis was applied to three cohort studies while the GATE assessment was used to assess eight cross-sectional study designs.

Children and adolescents with low PA had increased risk of new-onset asthma, and some showed a higher risk of current asthma/or wheezing, but we found variations among the studies.

## Discussion

The cohort studies showed that the overall risks of new-onset asthma/or wheezing increased up to 35 % in children with low PA, and three cross-sectional studies showed significant positive associations with low PA. Of the 11 studies we reviewed, more than 50 % suggested positive associations between childhood asthma and low PA. The critical problem was variation across the reviewed studies. We therefore applied appropriate epidemiological methods when performing meta-analysis of similar studies and when graphically assessing those that were dissimilar.

This systematic review followed established guidelines [29]. The review included >500 cases of new-onset (incident) asthma/or wheezing and approximately 4,000 current (prevalent) asthma cases. Although the number of studies was moderate, the inclusion of a variety of study designs may be advantageous. Previous investigations have produced contradictory results for the association under study, and the cross-sectional study design has limitations with respect to ruling out the directions of associations; therefore, we sought to identify studies with a longitudinal design. The longitudinal design of cohort studies overcomes the limitations of the cross-sectional design because measures of cause and effect are separated in time. Reverse causation (i.e., the notion that asthma causes low PA) was accounted for by the cohort studies [45, 51, 52]. For example, one study [45] included only asymptomatic children. Hence, we were able to derive some assessment of the directions of the associations. Other authors [58, 59] have proposed hypotheses fairly similar to ours; this review could confirm significant positive associations described by three [22–24] cross-sectional and two longitudinal [45, 52] studies.

The intensity of leisure-time activity studied by Vogelberg et al. [52] was similar to that of organized team sports studied by Islam et al. [51]. Although leisure-time activity differs from organized sports [37], they both fall along a spectrum of aerobic activities. The leisure activities included, e.g., running, bicycling, and swimming [52, 60], and the team sports encompassed a range of intensity from low to high [51, 61]. Thus, we could not identify systematic deviations in the PA definitions of these two studies [51, 52].

Generally, the quality of the reviewed studies was high. Although GATE does not provide one single quantitative assessment score [62], the observational studies appeared to reflect good standards for internal validity. We excluded ecological studies in an effort to retrieve studies with a rigorous design [63]. Although the clinical application of reviews is often overlooked [47], our results appear to align with those of others who have acknowledged the clinical importance of observational studies [64].

Recent systematic reviews have investigated the prevalence of wheezing in children [65] or PA in adolescents [66], but few have reviewed both. The asthma diagnosis was critical for our results. Asthma is a heterogeneous clinical syndrome [67], and because the diagnosis of asthma in children lacks a gold standard, it is ideally verified by uniform guidelines [68]. The asthma definitions in the current review were relatively uniform. Seven of the eleven studies used physician-confirmed asthma diagnoses, and our review populations were homogeneous (Europe and North America). Earlier reviews [65] that had to rely on less rigorous asthma symptom reports lack these characteristics.

All reviewed studies performed PA quantification. The cross-national survey, for example, used the ISAAC questionnaire [23] and showed a significant association between asthma and low PA in adolescents but not in children. Data on activity in young children are often difficult for parents to report, and in fact, some of the cross-sectional studies included 6-year-olds. The younger children in these studies received parental assistance with the questionnaire, and thus we cannot rule out information bias. Recent evidence has certainly suggested that parents and peers influence PA in both healthy [69–71] and asthmatic [72] children. Although we recognize that accelerometry still requires technical improvements for optimal use in the youngest children [71, 73, 74], the reported findings appear to align with earlier objective measurements that employed accelerometry [59].

The strength of the effect sizes varied, and the smaller studies [22, 24] yielded larger effect sizes, as expected. Moreover, low PA varied; for example, Lang et al. [22] analyzed daily PA durations as low as 30 min. Analogously, Nystad et al. [9] quantified “very low” PA (<1 h/week). Lang et al. [22] measured PA during the school day, whereas Nystad et al. [9] studied PA outside of school hours. This diurnal variation in PA could be of importance to the results because energy expended during seated school-day activities varies from that expended during leisure PA [75]. The reporting of PA also varied and included duration and frequency. Although protective associations between PA and current asthma were non-significant, Nystad [55] suggested that these associations could be a factor when PA frequency is analyzed. Therefore, although PA frequency and duration show correlations in children [76], it may be relevant to report both.

Our review may have certain limitations. Formal meta-analysis of the cross-sectional studies was not reasonable given that the overestimation of effects is well documented [57]. Although GATE is only one of a number of existing quality appraisal tools [62], we acknowledge that it provided some systematization to our assessments.

Limitations of the calculations were also made evident when individual patient data were not provided in the articles. We lacked some data on the exposed and non-exposed groups described in the cross-sectional studies. Although statistical methodology exists for imputing data [47], no such technique was used in the current analyses. Our meta-analysis was a two-stage process [48]. The first results produced were the summary statistics of each individual study that was included. These results agreed with the conclusions of each study. Because the meta-analysis inclusion criteria were met, we then combined the statistics. We have discussed the variation in the exposures (PA) and outcomes (asthma/or wheezing) of the meta-analyzed studies [45, 51, 52], but the basic cohort methodologies also appeared to be rather similar.

The meta-analysis revealed increased risks of new-onset asthma among children who reported low PA. The funnel plot showed that these three studies lay within the confidence intervals; this illustration may favor limited heterogeneity. Although we must expect some inter-study variability, the random-effects model could have assigned disproportionate influence to the studies with the smallest sample sizes. We cannot draw firm conclusions from the limited number of cohort studies available, but the parallel results for the fixed and random estimations may indicate only modest heterogeneity. We generated I-squared values of approximately 60 % (with non-significant chi-squared tests), and based on current guidelines [77], these findings may support our assumptions regarding heterogeneity. In the random- and fixed-effects models, this result implied that 60 % of the between-study heterogeneity could be explained by true study variation [47, 78].

Body composition was not reviewed in this study. Although obesity is related to childhood asthma [79, 80], the effects of asthma and weight on lung function are highly variable [81].

Future studies should involve the participation of clinical professionals. Clinicians (e.g., pediatricians or epidemiologists) may find our results useful when inquiring about the PA of their young patients who present with respiratory symptoms or asthma.

## Conclusions

Of the 1,571 titles reviewed, we analyzed 11 original articles. Overall, we observed indications that children who were physically inactive may have a higher risk of asthma/or wheezing compared with active children. This review also revealed a critical need for future longitudinal assessments of low PA, its mechanisms, and its implications for incident asthma in children.

## Additional files

Additional file 1: PRISMA Items Used in Reporting in the Current Systematic Literature Review. Additional file 1 presents the PRISMA checklist items that were examined, with the draft article page numbers. (DOCX 29 kb) Additional file 2: Reporting Items of the Meta-analysis Of Observational Studies in Epidemiology (MOOSE) Group. (PDF 532 kb) Additional file 3: A. Articles Excluded (*n* = 54) from the Systematic Review Grouped by Exclusion Rationale. B. Articles Excluded (*n* = 7) from the Systematic Review after Review of the Reference Lists. (DOCX 25 kb) Additional file 4: Data from Eight Non-Meta-Analyzed Studies Extracted to the GATE Calculator and the GATE-Lite Appraisal Forms. (PDF 8281 kb)

## Abbreviations

BHR: bronchial hyperresponsiveness/or bronchial hyperresponsibility
BMI: body mass index
CI: confidence interval
EIA: exercise-induced asthma
GATE: Graphic Appraisal Tool for Epidemiology
HR: hazard ratio
ISAAC: International Study of Asthma and Allergies in Childhood
MeSH: medical subject heading
MET: metabolic equivalent
OR: odds ratio
PA: physical activity
RCT: randomised controlled trial
SE: standard error
SES: socio-economic status
