# Supplementary material for: Childhood asthma and physical activity: a systematic review with meta-analysis and Graphic Appraisal Tool for Epidemiology assessment
Source: BMC Pediatr. 2016 Apr 18;16:50. doi: 10.1186/s12887-016-0571-4 (PMC4836150; doi:10.1186/s12887-016-0571-4)
Supplement: Additional file 2: — Reporting Items of the Meta-analysis Of Observational Studies in Epidemiology (MOOSE) Group. (PDF 532 kb) [file 12887_2016_571_MOESM2_ESM.pdf]

The JAMA Network

From: **Meta-analysis of Observational Studies in Epidemiology: A Proposal for Reporting**

JAMA. 2000;283(15):2008-2012. doi:10.1001/jama.283.15.2008 [31]

Table. A Proposed Reporting Checklist for Authors, Editors, and Reviewers of Meta-analyses of Observational Studies

	Reporting of background should include
x	Problem definition
x	Hypothesis statement
x	Description of study outcome(s)
x	Type of exposure or intervention used
x	Type of study designs used
x	Study population
	Reporting of search strategy should include
x	Qualifications of searchers (eg, librarians and investigators)
x	Search strategy, including time period included in the synthesis and keywords
x	Effort to include all available studies, including contact with authors
x	Databases and registries searched
x	Search software used, name and version, including special features used (eg, explosion)
x	Use of hand searching (eg, reference lists of obtained articles)
x	List of citations located and those excluded, including justification
N/A	Method of addressing articles published in languages other than English
x	Method of handling abstracts and unpublished studies
x	Description of any contact with authors
	Reporting of methods should include
x	Description of relevance or appropriateness of studies assembled for assessing the hypothesis to be tested
x	Rationale for the selection and coding of data (eg, sound clinical principles or convenience)
x	Documentation of how data were classified and coded (eg, multiple raters, blinding, and interrater reliability)
x	Assessment of confounding (eg, comparability of cases and controls in studies where appropriate)
x	Assessment of study quality, including blinding of quality assessors; stratification or regression on possible predictors of study results
x	Assessment of heterogeneity
x	Description of statistical methods (eg, complete description of fixed or random effects models, justification of whether the chosen models account for predictors of study results, dose-response models, or cumulative meta-analysis) in sufficient detail to be replicated
x	Provision of appropriate tables and graphics
	Reporting of results should include
x	Graphic summarizing individual study estimates and overall estimate
x	Table giving descriptive information for each study included
N/A	Results of sensitivity testing (eg, subgroup analysis)
x	Indication of statistical uncertainty of findings
	Reporting of discussion should include
x	Quantitative assessment of bias (eg, publication bias)
x	Justification for exclusion (eg, exclusion of non–English-language citations)
x	Assessment of quality of included studies
	Reporting of conclusions should include
x	Consideration of alternative explanations for observed results
x	Generalization of the conclusions (ie, appropriate for the data presented and within the domain of the literature review)
x	Guidelines for future research
x	Disclosure of funding source

Figure Legend:

x : Reporting check point included in current review

N/A: Point not applicable to current review

Date of download: 9/19/2014

Copyright © 2014 American Medical Association. All rights reserved.
