# Supplementary material for: Childhood asthma and physical activity: a systematic review with meta-analysis and Graphic Appraisal Tool for Epidemiology assessment
Source: BMC Pediatr. 2016 Apr 18;16:50. doi: 10.1186/s12887-016-0571-4 (PMC4836150; doi:10.1186/s12887-016-0571-4)
Supplement: Additional file 3: — A. Articles Excluded (n = 54) from the Systematic Review Grouped by Exclusion Rationale. B. Articles Excluded (n = 7) from the Systematic Review after Review of the Reference Lists. (DOCX 25 kb) [file 12887_2016_571_MOESM3_ESM.docx]

**Additional file 3A.** Articles excluded (n=54) from the systematic review grouped by exclusion rationale^¶^.

| **Exclusion rationale** | **N** | **Reference** |
| --- | --- | --- |
| Young adults and asthma | 2 | Rasmussen et al., 2000; Beckett et al., 2001 |
| Adult onset asthma | 1 | Huovinen et al., 2001 |
| Fitness, body fat, asthma symptoms | 2 | Pianosi & Davis, 2004; Berntsen et al., Aug 2009 |
| EIA and PA | 10 | Varray et al., 1995; Ulrik & Backer, 1996; Baba et al., 1997; Homnich & Marks, 1998; Godfrey et al., 1999; MacAuley et al., 1999; Counil et al., 2001; Timonen et al., 2002; Haverkamp et al., 2005; Basaran et al., 2006 |
| Methodology report | 1 | Priftis et al., 2007 |
| Validation | 1 | Floro et al., 2009 |
| EIA and training recommendation | 1 | Small & Bar-Or, 1995 |
| Outcome EIA | 1 | Nolan, 1996 |
| EIA and fitness | 1 | Kitsantas & Zimmerman, 2000 |
| PA or fitness | 19 | George, 1996; Trost et al., 1998; Washington, 1999; LeMura et al., 2001; Eisenmann & Malina, 2002; Puyau et al., 2002; Rump et al. 2002; Beets & Pitetti, 2004; Cradock et al., 2004; King et al., 2004; Ozcelik et al., 2004; Rowlands et al., 2004; Stephens & Paridon, 2004; Macfarlane & Tomkinson, 2007; Tomkinson & Olds, 2007; Tomkinson, 2007; Ortega et al., 2008; Casazza et al., 2009; Morrow et al., 2009 |
| Low PA excluded (METS <4) | 1 | Ownby et al., 2007 |
| Exercise intervention (leucocyte outcome) | 1 | Schwindt et al., 2007 |
| Exercise intervention (lymphocyte outcome) | 1 | Rosa et al., 2009 |
| Medical intervention | 2 | Thio et al., 1996; Vahlkvist & Pedersen, 2009 |
| Physical self-concept and asthma | 1 | Chiang et al., 2005 |
| BHR | 1 | Nystad et al., 2001 |
| Data collected for other purpose, i.e., youth sleep patterns | 1 | Tsai et al., 2012 |
| Review | 2 | Welsh et al., 2004; Williams et al., 2008 |
| Other outcome/results |  |  |
| Activity counts | 2 | Eijkemans et al., 2008; Berntsen et al., Mar 2009 |
| Metabolic cost | 1 | Frost et al., 2002 |
| FENO | 1 | Gabriele et al., 2005 |
| Disease control | 1 | Fanelli et al., 2007 |

¶ When the exclusion rationale was not stated in the exclusion criteria, the reason for exclusion is provided in parentheses.

BHR: Bronchial hyperresponsiveness; EIA: Exercise-induced asthma; FENO: Fractional exhaled nitric oxide; N: Number; PA: Physical activity.

**Additional file 3B.** Articles excluded (n=7) from the systematic review after review of reference lists, grouped by exclusion rationale^¶^.

| **Exclusion rationale** | **N** | **Reference** |
| --- | --- | --- |
| Fitness | 1 | Garfinkel et al., 1992 |
| Cumulative incidence of asthma | 1 | Selsnes et al., 2002 |
| Oxidant injury | 1 | Ercan et al., 2006 |
| EIA pathophysiology and treatment | 1 | Wilber, 2002 |
| Asthma subjects excluded | 1 | Rasmussen et al., 1999 |
| Asthma subjects (approx. percent reported for n) | 1 | Tsai et al., 2007 |
| Asthma and obesity | 1 | Gilliland et al., 2003 |

¶ When the exclusion rationale was not stated in the exclusion criteria, the reason for exclusion is provided in parentheses.

Approx.: Approximate; EIA: Exercise-induced asthma; N: Number.
