# Supplementary material for: Childhood asthma and physical activity: a systematic review with meta-analysis and Graphic Appraisal Tool for Epidemiology assessment
Source: BMC Pediatr. 2016 Apr 18;16:50. doi: 10.1186/s12887-016-0571-4 (PMC4836150; doi:10.1186/s12887-016-0571-4)
Supplement: Additional file 4: — Data from Eight Non-Meta-Analyzed Studies Extracted to the GATE Calculator and the GATE-Lite Appraisal Forms. (PDF 8281 kb) [file 12887_2016_571_MOESM4_ESM.pdf]

GATE Calculator - Risk Factor Cohort Studies

Step 3: Appraise study using PECOT framework (fill in this Calculator in conjunction with appropriate GATE CAT)

a. "hang" the study numbers on the GATE (Graphic Appraisal Tool for Epidemiology) Frame

Assessed by:	LL	Assessed when:	12-Nov-14	Publication details:	Nystad, 1997

Notes for use:

Enter study numbers in yellow areas. Help notes appear in moveable boxes.

Enter study descriptions in pink areas

The form calculates results and displays them in the green areas below.

If performing multiple analyses (e.g. reporting on more than one exposure) select tabs 'Analysis 2', 'Analysis 3', etc from the bottom left of the screen

Use together with page 2 of the GATE CAT Risk Factor Cohort Studies form

Populations

Study Setting

Eligible population

Participant population: 4021

Participant subgroup:

Exposure & Comparison

	Exposure factor (EG)	(CG) Comparison factor
	**Physical activity1-3hrs/wk**	Physical activity <=0.5h/wk
**Numbers allocated to EG & CG:**		
**Follow-up:**		
completed follow-up:	2053	582
drop-outs / lost during follow-up:		
**Percentage lost to follow up:**		

Outcomes

Outcome

If categorical.... what e.g. death? Current (prevalent) asthma

	EG	CG
participants with outcome:	a: 85	b: 30
without outcome:	c:	d:

If numerical.... what e.g. BP? **Outcome**:

	EG	CG
mean:		
standard deviation:		
or, standard error:		

Report results per (e.g. per 100): 100 persons

Results (unadjusted) with 95 % confidence intervals

Z-score: 1.96

Calculated in GATE frame

	Occurrence per 100 persons: in exposure group (EGO)	Occurrence per 100 persons: in comparison group (CGO)	Exposure effects per 100 persons: Relative effect (EGO/CGO)	Exposure effects per 100 persons: Absolute effect (EGO-CGO)	Number needed to expose (NNE) to prevent/cause 1 event
Categorical outcome: ntention to follow-up analyses					
95% CIs					
Categorical outcome: Completed f/u analyses	4.14	5.15	0.80	-1.01	-99
95% CIs	3.36 to 5.09	3.63 to 7.26	0.54 to 1.21	-3.01 to 0.98	-33 to ∞ to 102
Numerical outcome: Analysis of means					
95% CIs					

Please contribute your comments and suggestions on this form to: rt.jackson@auckland.ac.nz

Downloadable from www.epiq.co.nz
Copyright © 2004 Rod Jackson, University of Auckland

GATE-lite for RCTs & Observational (risk, prognosis, x-sectional) Studies 2012 and 2013

Study details: Nystad, 1997

STUDY QUESTION & DESIGN: describe with PECOT

P = Participants:

Describe:

- Setting: Three country districts
  Cross-sectional, Scandinavia (Norway)
- Eligibility criteria: School-children 1st-9th grade, age range 7-16 years
- Recruitment process:
  District I: Random selection of school classes
  District II and III: All school classes in 1st-9th grade
- % of eligibles who participated:
  DI: 85% (2188); DII: 89% (1045); DIII: 95% (788)

EG = Exposed Group [Intervention/Risk factor]

Method of allocation

Describe E (how measured if not RCT)

Physical activity (1-3 hours per week) outside school hours, sports, or exercise which made child get out of breath or sweaty. From study HBSC (Health Behaviour in School-aged Children), WHO coordinated questionnaire

WHO: World Health Organisation

CG = Comparison Group [Control/comparison]

Describe C (how measured if not RCT)

Physical activity ≤ 0.5 hour per week

O = Outcomes: Primary (& 2° include adverse)
T = Time when outcomes counted (at what point in time or over what time period)

Describe O & T: how / when measured

O: Current (prevalent) asthma using ISAAC (International Study of Asthma and Allergies in Childhood) questionnaire plus question on current asthma from article reference 15: Skarpaas & Gulsvik, 1985

T: Jan-Nov 1994

STUDY NUMBERS: hang on GATE frame

Setting

Eligibles

n = _4585_

P

n = _4021_

Allocated: randomly or by measurement

EG Allocated =_ _ _ _ _ CG Allocated =_ _ _ _ _

EG | CG

EG completed follow-up (f/u) =_2053_ _

CG completed f/u =_582_ _

EG incomplete f/u =_ _ _ _ _ CG incomplete f/u =_ _ _ _

	EG	CG
+	a = 85	b = 30
-	c	d

STUDY ERROR: assess using RAMBOMAN

Recruitment appropriate to study goals?

Setting/eligible population appropriate, given study goals? Yes

Participants representative of Eligibles? -

Participant risk/prognostic profile reported? Yes

Allocation (± adjustment) to EG & CG successful/done accurately?

If allocated randomly: Was process concealed? Were EG&CG similar? -

If allocated by measurement: Was it done accurately? Done before outcomes? Were differences between EG&CG documented? N/A

Maintenance of EG & CG as allocated sufficient?

Compliance high, Contamination low? Yes, N/A

Co-interventions similar in EG&CG? N/A

Completeness of follow-up high? Yes

Participants/Investigators blind to Exp. status? No

Blind and Objective Measurements?

Outcomes measured accurately? Yes

STUDY ANALYSES

Outcome & Time	EGO = a/EG	CGO = b/CG	RR = EGO/CGO ± 95% CI	RD = EGO-CGO ± 95% CI	NNT = 1/RD ± 95% CI
Reported results per 100 persons, please refer to front calculator page	4.14	5.15	0.80	-1.01	-99

ANalyses: Intention to treat (if RCT)?______ Adjusted if EG & CG different?______ 95% CI or p-values given?______

Summary:

1. **Non-random error sufficiently low?** (AMBOM: amount & direction of bias):
   Differences between asthma and controls further depended on district (area) in this study. There was variation in prevalence of e.g., current asthma by district
2. **Analytical error sufficiently low?** (AN: ITT /adjusted analyses):
   Possibly ok, analyses adjusted for: Age, gender, study area
3. **Random error sufficiently low?** (95% CIs: and if no statistically significant effects demonstrated was study power/sample size sufficiently high): Likely, ok (please refer to 95% CIs for EGO and CGO previous calculator page). The 95% CIs of EGO were narrow due to relatively large number of children (2053) exposed (1-3 hours per week) i.e. large denominator
4. **Size of effects sufficient to be meaningful?** (RR &/or RD): EGO/CGO possibly meaningful (slightly protective effect) based on the numbers described above and maybe in alignment with adjusted result reported by Nystad et al., 1997: OR(95%CI) between current asthma and PA 1-3 hours per week versus ≤0.5 hour per week (reference group): 1.0 (0.6, 1.5)
5. **If 1-4 ok, are findings applicable in practice?** (R): Results may be influenced by district variation but they may still be important and applicable to future research i.e. hypothesis generation as well as to clinical practice and regional health strategies

GLOSSARY

Use this form for questions about: interventions (RCTs & cohort studies), risk factors/causes (cohort & cross-sectional studies) or prognosis (cohort studies)

Hang the study on the GATE Frame

STUDY QUESTIONS/DESIGN: use PECOT to define study question & describe study design

Setting of study: Timing & locations in which Eligibles identified (e.g. country/urban/hospital).

Eligibles: those from study Setting who meet eligibility (i.e. inclusion / exclusion) criteria.

How were Eligibles identified from study setting: what kind of list (sampling frame) was used to identify potential participants: (e.g. hospital admission list, electoral rolls, advertisements).

P: Participants: recruited from Eligibles & allocated to EG/CG. How recruited from Eligibles (eg. randomly, consecutive)?

EG: Exposure Group: participants allocated to the main exposure (or intervention or prognostic group) being studied. If there are multiple exposures, use a new GATE frame for each exposure.

CG: Comparison Group: participants allocated to alternative (or no) exposure (i.e. control).

Outcome: specified study outcome(s) for analyses. If multiple outcomes, use additional GATE frames.

Time: when outcomes measured; at one point in time → (prevalence) or over a period of time ↓(incidence).

STUDY VALIDITY (non random error or bias): use RAMBOM to identify possible non random errors

Recruitment (mainly about external validity): were setting/Eligibles appropriate given the study goals &/or the reviewer's interests? If relevant, were participants representative of Eligibles? Could the results be generalised to relevant populations? This should be able to be determined from risk factor/prognostic profile of participants. In prognostic studies – were participants at similar stage in progression of their disease or condition?

Allocation: how well were participants allocated to E&C? If a trial were they **randomised** to E&C?

- If randomised, was allocation concealed (i.e. knowledge of group (EG or CG) participants allocated to concealed from staff & participants until after allocation documented)? Was randomization successful (i.e. EG & CG similar after randomisation – were baseline characteristics similar in each group)?
- If not randomised (observational study) were measurements of E&C accurate & done similarly for EG & CG? Were differences between EG & CG documented.

Maintenance: did participants remain in the groups (EG or CG) they were initially allocated to? Compliance: % participants allocated to EG (or CG) who remained exposed to E (or C) during study? Contamination: % participants allocated to CG who crossover to EG (& visa versa if CG an exposure)? Co-intervention: other significant interventions received unequally by EG&CG during follow-up? Completeness of follow-up: was it high & similar in EG & CG? Blinding: were participants / investigators blind to whether participants were exposed to E or C?

Blind Measurement of outcomes: were outcome assessors unaware if participants in EG or CG? **or**

Objective Measurement of outcomes. eg. based on biopsies; automated tests, x-rays, validated questionnaires?

STUDY ANALYSES (estimates of occurrence [EGO & CGO], effect sizes [RR & RD]) and random error [95% CI]

Intention to treat (or expose) analyses: did analyses (i.e. calculation of EGO & CGO) include all participants allocated to EG & CG, including anyone who dropped out during study or did not complete follow-up)?

Adjusted analyses (for confounders): Were EG & CG similar at baseline? If not, were analytical methods used to adjust for any differences, e.g. stratified analyses, multiple regression?

EGO: Exposure Group Occurrence (either incidence or prevalence measures; also known as Experimental Event Rate (EER) in RCTs). **CGO:** Comparison Group Occurrence (or Control Event Rate (CER) in RCTs). Most studies report **cumulative incidence** or **prevalence** measures of occurrence and EGO = a/EG & CGO = b/CG, and you should document over what time period (cumulative incidence) or at what point in time (prevalence) EGO & CGO are measured. However if EGO & CGO are calculated as **incidence rates** per unit time (T) (e.g. per year), then EGO = a/(EG x T) & CGO = b/(CG x T). EG x T and CG x T describe 'person-time.'

Effect estimates (measures for comparing EGO & CGO): Risk Ratio (RR) = EGO/CGO; more commonly known as Relative Risk. Odds Ratios & Hazards ratios are similar to RR. **Risk Difference (RD) = EGO-CGO;** also known as absolute risk difference. **NNT (or NNE) = 1/RD;** the number Needed to Treat (or expose) to change the number of outcomes by one (in a specified time). NNT(B): if exposure/intervention BENEFICIAL. NNT(H): if exposure/intervention HARMFUL. Note: NNT(H) often called NNH.

Random error in estimates of EGO, CGO, RR, RD & NNT/E is assessed by width of confidence interval (CI). A wide CI (i.e. big gap between upper & lower confidence limits (CL) = more random error = less precision.

STUDY SUMMARY

Non-random error (bias): what was the likely amount & direction of bias: is bias likely to substantially increase or decrease the observed difference between EGO & CGO (and therefore the effect sizes)?

Random error: would you make a different decision if the real effect was closer to upper CL than lower CL?

Power: if the effect sizes were not statistically significant, was study just too small to show meaningful effects?

Effect sizes: was the magnitude of the RR or RD (or NNT) sufficient to be meaningful/useful in practice?

Applicability: if effect sizes meaningful & errors small, are the findings likely to be applicable in practice?

REFERENCE: Jackson et al. The GATE frame: critical appraisal with pictures. In: Evidence-Based Medicine. 2006;11;35-38. Also in: Evidence-Based Nursing 2006; 9: 68-71, and in ACP Journal Club 2006; 144: A8-A11.

GATE Calculator - Risk Factor Cohort Studies

Step 3: Appraise study using PECOT framework (fill in this Calculator in conjunction with appropriate GATE CAT)

a. "hang" the study numbers on the GATE (Graphic Appraisal Tool for Epidemiology) Frame

Assessed by:	LL	Assessed when:	12-Nov-14	Publication details:	**Nystad et al., 2001**

Notes for use:

Enter study numbers in yellow areas.
Help notes appear in moveable boxes.

Enter study descriptions in pink areas

The form calculates results and displays them in the green areas below.

If performing multiple analyses (e.g. reporting on more than one exposure) select tabs 'Analysis 2', 'Analysis 3', etc from the bottom left of the screen

Use together with page 2 of the GATE CAT Risk Factor Cohort Studies form

Populations

Study Setting

Eligible population

Participant population: 2112

Participant subgroup:

Exposure & Comparison

Exposure factor (EG): **Physical activity<=1h/wk**

(CG) **Comparison factor**: Physical activity "none"

	EG	CG
**Numbers allocated to EG & CG:**		
**Follow-up:**		
completed follow-up:	517	238
drop-outs / lost during follow-up:		
**Percentage lost to follow up:**		

Outcomes

Outcome

If categorical.... what e.g. death? Wheeze and current asthma

	a (EG)	b (CG)
participants with outcome:	32	12
without outcome:		
	c	d

If numerical.... what e.g. BP? **Outcome**

	EG	CG
mean:		
standard deviation:		
or, standard error:		

Report results per (e.g. per 100): 100 persons

Results (unadjusted) with 95 % confidence intervals

Z-score: 1.96

Calculated in GATE frame

	Occurrence per 100 persons: in exposure group (EGO)	Occurrence per 100 persons: in comparison group (CGO)	Exposure effects per 100 persons: Relative effect (EGO/CGO)	Exposure effects per 100 persons: Absolute effect (EGO-CGO)	Number needed to expose (NNE) to prevent/cause 1 event
Categorical outcome: ntention to follow-up analyses					
95% CIs					
Categorical outcome: Completed f/u analyses	6.19	5.04	1.23	1.15	87
95% CIs	4.42 to 8.61	2.91 to 8.60	0.64 to 2.34	-2.32 to 4.62	-43 to ∞ to 22
Numerical outcome: Analysis of means					
95% CIs					

Please contribute your comments and suggestions on this form to: rt.jackson@auckland.ac.nz

Downloadable from www.epiq.co.nz
Copyright © 2004 Rod Jackson, University of Auckland

GATE-lite for RCTs & Observational (risk, prognosis, x-sectional) Studies 2012 and 2013

Study details: Nystad et al., 2001

STUDY QUESTION & DESIGN: describe with PECOT

P = Participants:

Describe:
- Setting: Cross-sectional, Scandinavia (Norway)
- Eligibility criteria: School-children, age range 7-16 years
- Recruitment process: School survey conducted in capital Oslo
- % of eligibles who participated: 95 5%

EG = Exposed Group [Intervention/Risk factor]

Method of allocation

Describe E (how measured if not RCT)

Physical activity (≤ 1 hour per week) outside school hours, sports, or exercise which made child get out of breath or sweaty. From study HBSC (Health Behaviour in School-aged Children), WHO coordinated questionnaire

WHO: World Health Organisation

CG = Comparison Group [Control/comparison]

Describe C (how measured if not RCT)

Physical activity "none" (hours per week)

O =Outcomes: Primary (& 2° include adverse)
T = Time when outcomes counted (at what point in time or over what time period)

Describe O & T: how / when measured

O: Wheeze past 12 months, data collected using ISAAC (International Study of Asthma and Allergies in Childhood) questionnaire
Plus question still (i.e. current) asthma using 1976 ATS-MRC (American Thoracic Society and Medical Research Council) questionnaire

T: 1994

STUDY NUMBERS: hang on GATE frame

Setting

Eligibles

n =_2188 ____

P

n = _2112_

Allocated: randomly or by measurement

EG Allocated =_____ **CG Allocated** =_____

EG | **CG**

EG completed follow-up (f/u) =_517__ | CG completed f/u =_238__

EG incomplete f/u =_____ | CG incomplete f/u =____

+	a = 32	b = 12
-	c	d

STUDY ERROR: assess using RAMBOMAN

Recruitment appropriate to study goals?

Setting/eligible population appropriate, given study goals? Yes

Participants representative of Eligibles? Yes

Participant risk/prognostic profile reported? Yes

Allocation (± adjustment) to EG & CG successful/done accurately?

If allocated randomly: Was process concealed? Were EG&CG similar? -

If allocated by measurement: Was it done accurately? Done before outcomes? Were differences between EG&CG documented? N/A

Maintenance of EG & CG as allocated sufficient?

Compliance high, Contamination low? Yes, N/A

Co-interventions similar in EG&CG? N/A

Completeness of follow-up high? Yes

Participants/Investigators blind to Exp. status? No

Blind and **Objective Measurements?**

Outcomes measured accurately? Yes

STUDY ANALYSES

Outcome & Time	EGO = a/EG	CGO = b/CG	RR = EGO/CGO ± 95% CI	RD = EGO-CGO ± 95% CI	NNT = 1/RD ± 95% CI
Reported results per 100 persons, please refer to front calculator page	6.19	5.04	1.23	1.15	87

ANalyses: Intention to treat (if RCT)?______ Adjusted if EG & CG different?______ 95% CI or p-values given?______

Summary:

1. **Non-random error sufficiently low?** (AMBOM: amount & direction of bias): Yes, this is likely as the study used validated methodologies and variation between groups of asthmatics and non-asthmatic children and between asthma subgroups have been accounted for
2. **Analytical error sufficiently low?** (AN: ITT /adjusted analyses) Possibly ok, analyses adjusted for: Age, atopy (eczema and /or hay fever), current asthma, gender
3. **Random error sufficiently low?** (95% CIs: and if no statistically significant effects demonstrated was study power/sample size sufficiently high): Likely, ok. CIs for EGO relatively narrow (front calculator page)
4. **Size of effects sufficient to be meaningful?** (RR &/or RD): Exposure effects (EGO/CGO and EGO-CGO) were not significant (CIs front calculator page) but due to a relatively large study population effects were possibly sufficiently large to be meaningful
5. **If 1-4 ok, are findings applicable in practice?** (R): These results may be important and applicable to future research i.e. hypothesis generating as well as to clinical practice. In a larger scale, if confirmed by others, these results may also assist national health policy strategy

GATE Calculator - Risk Factor Cohort Studies

Step 3: Appraise study using PECOT framework (fill in this Calculator in conjunction with appropriate GATE CAT)

a. "hang" the study numbers on the GATE (Graphic Appraisal Tool for Epidemiology) Frame

Assessed by:	LL	Assessed when:	12-Nov-14	Publication details:	Lang et al., 2004

Notes for use:

Enter study numbers in yellow areas. Help notes appear in moveable boxes.

Enter study descriptions in pink areas

The form calculates results and displays them in the green areas below.

If performing multiple analyses (e.g. reporting on more than one exposure) select tabs 'Analysis 2', 'Analysis 3', etc from the bottom left of the screen

Use together with page 2 of the GATE CAT Risk Factor Cohort Studies form

Populations

Study Setting

Eligible population

Participant population: 243

Participant subgroup

Exposure & Comparison

	Exposure factor (EG)	Comparison factor (CG)
	**Asthma ever&symptoms**	Control
**Numbers allocated to EG & CG:**		
**Follow-up:**		
completed follow-up:	137	106
drop-outs / lost during follow-up:		
**Percentage lost to follow up:**		

Outcomes

		(EG)	(CG)
**If categorical....** what e.g. death?	**Outcome**: Inactivity (<30 minutes/day)	a	b
	participants with outcome:	20	
	without outcome:	42	
		c	d
**If numerical....** what e.g. BP?	**Outcome**		
	mean:		
	standard deviation:		
	or, standard error:		

Report results per (e.g. per 100): 100 persons

Calculated in GATE frame

Results (unadjusted) with 95 % confidence intervals Z-score: 1.96

	Occurrence per 100 persons: in exposure group (EGO)	Occurrence per 100 persons: in comparison group (CGO)	Exposure effects per 100 persons: Relative effect (EGO/CGO)	Exposure effects per 100 persons: Absolute effect (EGO-CGO)	Number needed to expose (NNE) to prevent/cause 1 event
Categorical outcome: ntention to follow-up analyses					
95% CIs					
Categorical outcome: Completed f/u analyses	14.60				
95% CIs	9.65 to 21.47				
Numerical outcome: Analysis of means					
95% CIs					

Please contribute your comments and suggestions on this form to: rt.jackson@auckland.ac.nz

Downloadable from www.epiq.co.nz
Copyright © 2004 Rod Jackson, University of Auckland

GATE-lite for RCTs & Observational (risk, prognosis, x-sectional) Studies 2012 and 2013

Study details: Lang et al., 2004

STUDY QUESTION & DESIGN: describe with PECOT

P = Participants:

Describe:
- Setting: Urban, primary care pediatric clinic, cross-sectional, North America
- Eligibility criteria: Clinic enrolees, age range 6-12 years
- Recruitment process: Letters and phone calls. Data collected using questionnaire at phone interview
- % of eligibles who participated: 34% (overall interview completion rate for letters mailed) and 36% (of families eligible for phone contact)

EG = Exposed Group [Intervention/Risk factor]

Method of allocation

Describe E (how measured if not RCT)

Ever diagnosed with asthma by medical provider and if child had had some asthma symptoms in the last 12 months
Asthma severity classification used 4 steps (frequency of day and night symptoms past month) of National Asthma Education and Prevention Program guidelines

CG = Comparison Group [Control/comparison]

Describe C (how measured if not RCT)
Controls without asthma

O =Outcomes: Primary (& 2° include adverse)
T = Time when outcomes counted (at what point in time or over what time period)

Describe O & T: how / when measured
O: Inactivity (<30 minutes per day)
T. Late August and mid-December 2001

STUDY NUMBERS: hang on GATE frame

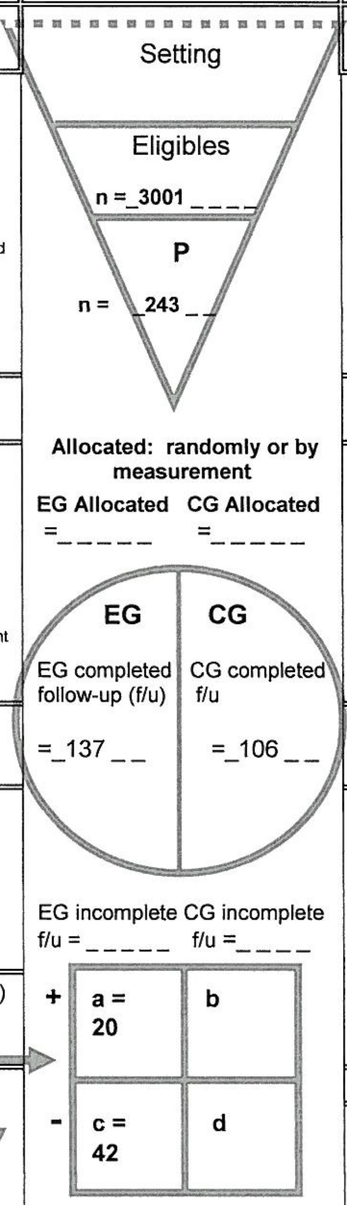

STUDY ERROR: assess using RAMBOMAN

Recruitment appropriate to study goals?

Setting/eligible population appropriate, given study goals? Yes

Participants representative of Eligibles? Somewhat, likely

Participant risk/prognostic profile reported? Yes

Allocation (± adjustment) to EG & CG successful/done accurately?

If allocated randomly: Was process concealed? Were EG&CG similar? -

If allocated by measurement: Was it done accurately? Done before outcomes? Were differences between EG&CG documented? Yes

Maintenance of EG & CG as allocated sufficient?

Compliance high, Contamination low? Yes, N/A

Co-interventions similar in EG&CG? N/A

Completeness of follow-up high? Yes

Participants/Investigators blind to Exp. status? Yes, until interview-response

Blind and **Objective Measurements?**

Outcomes measured accurately? Yes

STUDY ANALYSES

Outcome & Time	EGO = a/EG	CGO = b/CG	RR = EGO/CGO ± 95% CI	RD = EGO-CGO ± 95% CI	NNT = 1/RD ± 95% CI
Reported results per 100 persons, please refer to front calculator page	14.6				

ANalyses: Intention to treat (if RCT)?_______Adjusted if EG & CG different?_______95% CI or p-values given?_______

Summary:

1. **Non-random error sufficiently low?** (AMBOM: amount & direction of bias): N/A as data for CGO not given in article. Effect size moderate with wide CIs, possibly reflecting relatively small sample size
2. **Analytical error sufficiently low?** (AN: ITT /adjusted analyses): Possibly ok, analyses adjusted for: Gender, health beliefs: E.g., child can do as much physical activity as children similar age without asthma or child upset with strenuous activity
3. **Random error sufficiently low?** (95% CIs: and if no statistically significant effects demonstrated was study power/sample size sufficiently high): Possibly ok and number of participants close to pre-calculated sample size
4. **Size of effects sufficient to be meaningful?** (RR &/or RD): N/A
5. **If 1-4 ok, are findings applicable in practice?** (R): Based on points 1-3 these results may contribute data to clinical practice and be of importance and applicable to future research (i.e. hypothesis generating)

GATE Calculator - Risk Factor Cohort Studies

Step 3: Appraise study using PECOT framework (fill in this Calculator in conjunction with appropriate GATE CAT)

a. "hang" the study numbers on the GATE (Graphic Appraisal Tool for Epidemiology) Frame

Assessed by:	LL	Assessed when:	12-Nov-14	Publication details:	**Jones et al., 2006**

Populations

Study Setting

Eligible population

Participant population: 13553

Participant subgroup:

Notes for use:

Enter study numbers in yellow areas. Help notes appear in moveable boxes.

Enter study descriptions in pink areas

The form calculates results and displays them in the green areas below.

If performing multiple analyses (e.g. reporting on more than one exposure) select tabs 'Analysis 2', 'Analysis 3', etc from the bottom left of the screen

Use together with page 2 of the GATE CAT Risk Factor Cohort Studies form

Exposure & Comparison

	Exposure factor (EG)	Comparison factor (CG)
	**Current asthma yes**	No current asthma
**Numbers allocated to EG & CG:**		
**Follow-up:**		
completed follow-up:	1943	
drop-outs / lost during follow-up:		
**Percentage lost to follow up:**		

Outcomes

	EG	CG
**If categorical....** what e.g. death? Outcome: Moderate physical activity	a	b
participants with outcome:	486	
without outcome:	c	d
**If numerical....** what e.g. BP? Outcome:		
mean:		
standard deviation:		
or, standard error:		

Report results per (e.g. per 100): 100 persons

Results (unadjusted) with 95 % confidence intervals Z-score: 1.96

Calculated in GATE frame

	Occurrence per 100 persons: in exposure group (EGO)	in comparison group (CGO)	Exposure effects per 100 persons: Relative effect (EGO/CGO)	Absolute effect (EGO-CGO)	Number needed to expose (NNE) to prevent/cause 1 event
Categorical outcome: ntention to follow-up analyses 95% CIs					
Categorical outcome: Completed f/u analyses 95% CIs	25.01 (23.14 to 26.99)				
Numerical outcome: Analysis of means 95% CIs					

Please contribute your comments and suggestions on this form to: rt.jackson@auckland.ac.nz

Downloadable from www.epiq.co.nz
Copyright © 2004 Rod Jackson, University of Auckland

GATE-lite for RCTs & Observational (risk, prognosis, x-sectional) Studies 2012 and 2013

Study details: Jones et al., 2006

STUDY QUESTION & DESIGN: describe with PECOT	STUDY NUMBERS: hang on GATE frame	STUDY ERROR: assess using RAMBOMAN
**P = Participants**:	Setting	**Recruitment** appropriate to study goals?
Describe:<br>- Setting: Schools (private and public) participating in Youth Risk Behaviour Survey (YRBS) developed by Centers for Disease Control and Prevention (CDC). Cross-sectional, North America<br>- Eligibility criteria: Grades 9-12<br>- Recruitment process: Three-stage cluster sampling design, participants anonymous<br>- % of eligibles who participated: Overall, school, and student response rates 67%, 81%, 83%, respectively	Eligibles<br>n =______<br>P<br>n = _13553_	Setting/eligible population appropriate, given study goals? Yes<br>Participants representative of Eligibles? Yes<br>Participant risk/prognostic profile reported? Yes
**EG = Exposed** Group [Intervention/Risk factor]	Allocated: randomly or by measurement	**Allocation** (± adjustment) to EG & CG successful/done accurately?
Method of allocation<br>Describe E (how measured if not RCT):<br>Current asthma defined as<br>Was ever told by a doctor or nurse that student had asthma *and* during the12 months preceding the survey, student either had asthma but no episode of asthma or asthma attack or had an episode of asthma or asthma attack	EG Allocated =_____ CG Allocated =_____<br>EG CG<br>EG completed follow-up (f/u) =_1943__<br>CG completed f/u =_____	If allocated randomly: Was process concealed? Were EG&CG similar? -<br>If allocated by measurement: Was it done accurately? Done before outcomes? Were differences between EG&CG documented? N/A
**CG = Comparison** Group [Control/comparison]		**Maintenance** of EG & CG as allocated sufficient?
Describe C (how measured if not RCT)<br>Uncertainty in attempt to derive exact data on background population	EG incomplete f/u =_____<br>CG incomplete f/u =____	Compliance high, Contamination low? Yes, N/A<br>Co-interventions similar in EG&CG? N/A<br>Completeness of follow-up high? Yes<br>Participants/Investigators blind to Exp. status? Self-administered pc-scannable questionnaire applied
**O =Outcomes:** Primary (& 2° include adverse)<br>**T = Time** when outcomes counted (at what point in time or over what time period)	+ a = 486 | b<br>- c | d	**Blind** and **Objective Measurements?**
Describe O & T: how / when measured<br>O: Participation in sufficient moderate physical activity (PA)*<br>T: 2003<br>*PA not making student sweat and breathe hard for >= 30 minutes on >= 5 of the 7 days preceding the survey		Outcomes measured accurately? Yes

STUDY ANALYSES

Outcome & Time	EGO = a/EG	CGO = b/CG	RR = EGO/CGO ± 95% CI	RD = EGO-CGO ± 95% CI	NNT = 1/RD ± 95% CI
Reported results per 100 persons, please refer to front calculator page	25.01				

ANalyses: Intention to treat (if RCT)? ______Adjusted if EG & CG different?______95% CI or p-values given?_______

Summary:

1. **Non-random error sufficiently low?** (AMBOM: amount & direction of bias): Number in current asthma group (exposed) calculated from percentage (25.0%) provided in article's Table 3. For this review we have not recalculated numbers (24.1%) for comparison group
2. **Analytical error sufficiently low?** (AN: ITT /adjusted analyses): Possibly ok, analyses adjusted for: Grade, race/ethnicity, gender
3. **Random error sufficiently low?** (95% CIs: and if no statistically significant effects demonstrated was study power/sample size sufficiently high): Possibly an acceptable estimation, 95% CIs of occurrence (front calculator page) appear relatively narrow
4. **Size of effects sufficient to be meaningful?** (RR &/or RD): N/A
5. **If 1-4 ok, are findings applicable in practice?** (R): Points 1 and 4 not fully assessed, but these large-scale survey results, nonetheless, may contribute data of importance to future research (hypothesis generation), clinical practice, and health policy specific to North America

GATE Calculator - Risk Factor Cohort Studies

Step 3: Appraise study using PECOT framework (fill in this Calculator in conjunction with appropriate GATE CAT)

a. "hang" the study numbers on the GATE (Graphic Appraisal Tool for Epidemiology) Frame

Assessed by:	LL	Assessed when:	12-Nov-14	Publication details:	**Priftis et al., 2007**

Notes for use:

Enter study numbers in yellow areas. Help notes appear in moveable boxes.

Enter study descriptions in pink areas

The form calculates results and displays them in the green areas below.

If performing multiple analyses (e.g. reporting on more than one exposure) select tabs 'Analysis 2', 'Analysis 3', etc from the bottom left of the screen

Use together with page 2 of the GATE CAT Risk Factor Cohort Studies form

Populations

Study Setting

Eligible population

Participant population: 700

Participant subgroup

Exposure & Comparison

	**Exposure factor** (EG)	(CG) **Comparison factor**
	**No activity**	No activity last week
**Numbers allocated to EG & CG:**		
**Follow-up:**		
completed follow-up:		57
drop-outs / lost during follow-up:		
**Percentage lost to follow up:**		

Outcomes

	**Outcome**	EG	CG
**If categorical....** what e.g. death?	Asthma symptoms	a	b
participants with outcome:			10
without outcome:		c	47 d
**If numerical....** what e.g. BP?	**Outcome**		
mean:			
standard deviation:			
or, standard error:			

Report results per (e.g. per 100): 100 persons

Calculated in GATE frame

Results (unadjusted) with 95 % confidence intervals Z-score: 1.96

	Occurrence per 100 persons: in exposure group (EGO)	Occurrence per 100 persons: in comparison group (CGO)	Exposure effects per 100 persons: Relative effect (EGO/CGO)	Exposure effects per 100 persons: Absolute effect (EGO-CGO)	Number needed to expose (NNE) to prevent/cause 1 event
Categorical outcome: ntention to follow-up analyses 95% CIs					
Categorical outcome: Completed f/u analyses		17.54			
95% CIs		9.82 to 29.37			
Numerical outcome: Analysis of means 95% CIs					

Please contribute your comments and suggestions on this form to: rt.jackson@auckland.ac.nz

Downloadable from www.epiq.co.nz
Copyright © 2004 Rod Jackson, University of Auckland

GATE-lite for RCTs & Observational (risk, prognosis, x-sectional) Studies 2012 and 2013

Study details: Priftis et al., 2007

STUDY QUESTION & DESIGN: describe with PECOT	STUDY NUMBERS: hang on GATE frame	STUDY ERROR: assess using RAMBOMAN

P = Participants:

Describe:

- Setting: Children from 18 schools, context: PANACEA* study. Cross-sectional, Europe (Greece)
  *Physical activity, Nutrition and Allergies in Children examined in Athens
- Eligibility criteria: Grade 4-6, age range 10-12 years Male: 323 Premenstrual girls only
- Recruitment process: Schools randomly selected (from list of regional education offices)
- % of eligibles who participated: Overall participation rate 83.5%

Setting

Eligibles

n =_______

P

n = _700_

Recruitment appropriate to study goals?

Setting/eligible population appropriate, given study goals? Yes

Participants representative of Eligibles? -

Participant risk/prognostic profile reported? Yes

EG = Exposed Group [Intervention/Risk factor]

Method of allocation

Describe E (how measured if not RCT):

Data used for this Gate assessment:
Physical activity (PA) questionnaire response to: "Not participating in any physical activity" (PA) i.e. recreational, leisure activities such as brisk walking, running, swimming, cycling
Question was on: Number of times per week with accompanying shortness of breath for 20 min during leisure activities

Allocated: randomly or by measurement

EG Allocated =_____ **CG Allocated** =_____

EG | **CG**

EG completed follow-up (f/u): These data not provided in article

CG completed f/u =_57__

Allocation (± adjustment) to EG & CG successful/done accurately?

If allocated randomly: Was process concealed? Were EG&CG similar? -

If allocated by measurement: Was it done accurately? Done before outcomes? Were differences between EG&CG documented? N/A

CG = Comparison Group [Control/comparison]

Describe C (how measured if not RCT)

No participation in recreational activities last week

EG incomplete f/u = _____ CG incomplete f/u =____

Maintenance of EG & CG as allocated sufficient?

Compliance high, Contamination low? Yes, N/A

Co-interventions similar in EG&CG? N/A

Completeness of follow-up high? Yes

Participants/Investigators blind to Exp. status? No

O =Outcomes: Primary (& 2° include adverse)

T = Time when outcomes counted (at what point in time or over what time period)

Describe O & T: how / when measured

O: Asthma symptoms using ISAAC (International Study of Asthma and Allergies in Childhood) questionnaire. E.g.: Ever asthma, ever wheeze
T: Published 2007

	EG	CG
+	a	b = 10
-	c	d = 47

Blind and **Objective Measurements?**

Outcomes measured accurately? Yes

STUDY ANALYSE

Outcome & Time	EGO = a/EG	CGO = b/CG	RR = EGO/CGO ± 95% CI	RD = EGO-CGO ± 95% CI	NNT = 1/RD ± 95% CI
Reported results per 100 persons, please refer to front calculator page	-	17.54			

ANalyses: Intention to treat (if RCT)?______Adjusted if EG & CG different?______95% CI or p-values given?________

Summary:

1. **Non-random error sufficiently low?** (AMBOM: amount & direction of bias): Yes, this is possible. The study used a special validated questionnaire (article reference no. 14. Argiropoulou et al. J Sports Sci Med 2004; 3: 147-159) to record physical activities and the questions were read by the researcher to each participating child
2. **Analytical error sufficiently low?** (AN: ITT /adjusted analyses): Possibly ok, analyses adjusted for: Body weight (per 5 kg), time of TV-viewing or playing video games (per 1 hour)
3. **Random error sufficiently low?** (95% CIs: and if no statistically significant effects demonstrated was study power/sample size sufficiently high): The power of the study was possibly ok (in article text referral to power calculation ~80%). A significant effect size was indicated for boys by the 95% CI (above). Though, as discussed by article Authors, the relatively small sample size may have represented a limitation
4. **Size of effects sufficient to be meaningful?** (RR &/or RD): N/A
5. **If 1-4 ok, are findings applicable in practice?** (R): We were unable to extract specific data for point 4 but these survey results which are part of PANACEA population study may contribute data of importance to future research (hypothesis generation) as well as to clinical practice and health policy at national level

GATE Calculator - Risk Factor Cohort Studies

Step 3: Appraise study using PECOT framework (fill in this Calculator in conjunction with appropriate GATE CAT)

a. "hang" the study numbers on the GATE (Graphic Appraisal Tool for Epidemiology) Frame

Assessed by:	LL	Assessed when:	13-Nov-14	Publication details:	**Corbo et al., 2008**

Notes for use:

Enter study numbers in yellow areas.
Help notes appear in moveable boxes.

Enter study descriptions in pink areas

The form calculates results and displays them in the green areas below.

If performing multiple analyses (e.g. reporting on more than one exposure) select tabs 'Analysis 2', 'Analysis 3', etc from the bottom left of the screen

Use together with page 2 of the GATE CAT Risk Factor Cohort Studies form

Populations

Study Setting

Eligible population

Participant population: 20016

Participant subgroup

Exposure & Comparison

	**Exposure factor** (EG)	**Comparison factor** (CG)
	**Sports 1-2 times/week**	"None" (no regular sports)
**Numbers allocated to EG & CG:**		
**Follow-up:**		
completed follow-up:		
drop-outs / lost during follow-up:		
**Percentage lost to follow up:**		

Outcomes

If categorical.... what e.g. death? **Outcome**: Current asthma

	EG	CG
participants with outcome:	a 121	b 113
without outcome:	c	d

If numerical.... what e.g. BP? **Outcome**:

	EG	CG
mean:		
standard deviation:		
or, standard error:		

Report results per (e.g. per 100): 100 persons

Results (unadjusted) with 95 % confidence intervals

Z-score: 1.96

Calculated in GATE frame

	Occurrence per 100 persons: in exposure group (EGO)	Occurrence per 100 persons: in comparison group (CGO)	Exposure effects per 100 persons: Relative effect (EGO/CGO)	Exposure effects per 100 persons: Absolute effect (EGO-CGO)	Number needed to expose (NNE) to prevent/cause 1 event
Categorical outcome: ntention to follow-up analyses 95% CIs					
Categorical outcome: Completed f/u analyses 95% CIs					
Numerical outcome: Analysis of means 95% CIs					

Please contribute your comments and suggestions on this form to: rt.jackson@auckland.ac.nz

Downloadable from www.epiq.co.nz
Copyright © 2004 Rod Jackson, University of Auckland

GATE-lite for RCTs & Observational (risk, prognosis, x-sectional) Studies 2012 and 2013

Study details: Corbo et al., 2008

STUDY QUESTION & DESIGN: describe with PECOT

P = Participants:

Describe:

- Setting: Multicenter, population-based study SIDRIA-2 project in ISAAC# frame. Cross-sectional, Europe (Italy)
  # International Study of Asthma and Allergies in Childhood
- Eligibility criteria: Children in age range 6-7 years
- Recruitment process: Cluster sampling. SIDRIA-2 added random extraction of 50% of schools already enrolled in SIDRIA phase 1[1]
  1: Article reference no. 12: Galassi et al., Pediatrics 2006; 117: 34-42
- % of eligibles who participated: Response rate 89% "of the target"

EG = Exposed Group [Intervention/Risk factor]

Method of allocation

Describe E (how measured if not RCT):

Physical activity (PA) levels (1-2 times per week) using regular sports information (i.e. formal games or other forms of aerobic exercise*) collected via standardized questionnaire, self-administered, and parent-completed

*categories: none, rarely, 1-2 times per week, 3+ times per week

CG = Comparison Group [Control/comparison]

Describe C (how measured if not RCT)

PA "none" i.e. no regular sports

O =Outcomes: Primary (& 2° include adverse)
T = Time when outcomes counted (at what point in time or over what time period)

Describe O & T: how / when measured

O: Current asthma: Lifetime asthma and either asthma symptoms (wheeze, dyspnoea, morning chest tightness) during the last year or medical treatment for diagnosed asthma or a hospital admission for asthma in the last 12 months
T: Jan to May 2002

STUDY NUMBERS: hang on GATE frame

Setting

Eligibles
n =____

P
n = _20016_

Allocated: randomly or by measurement

EG Allocated =_____ **CG Allocated** =_____

EG **CG**

EG completed follow-up (f/u) CG completed f/u

Data not provided in article

EG incomplete f/u =_____ CG incomplete f/u =____

	EG	CG
+	a = 121	b = 113
-	c	d

STUDY ERROR: assess using RAMBOMAN

Recruitment appropriate to study goals?

Setting/eligible population appropriate, given study goals? Yes

Participants representative of Eligibles? Yes

Participant risk/prognostic profile reported? Yes

Allocation (± adjustment) to EG & CG successful/done accurately?

If allocated randomly: Was process concealed? Were EG&CG similar? -

If allocated by measurement: Was it done accurately? Done before outcomes? Were differences between EG&CG documented? N/A

Maintenance of EG & CG as allocated sufficient?

Compliance high, Contamination low? Yes, N/A

Co-interventions similar in EG&CG? N/A

Completeness of follow-up high? Yes

Participants/Investigators blind to Exp. status? No

Blind and **Objective Measurements?**

Outcomes measured accurately? Yes

STUDY ANALYSE

Outcome & Time	EGO = a/EG	CGO = b/CG	RR = EGO/CGO ± 95% CI	RD = EGO-CGO ± 95% CI	NNT = 1/RD ± 95% CI
Corbo et al., 2008, Table 4: Association [OR (95%CI)] between current asthma and low frequency of regular sports (1-2 times per week) vs none (reference group): 1.13 (0.93, 1.38) (*P* trend=0.069)	-				

ANalyses: Intention to treat (if RCT)?______ Adjusted if EG & CG different?______ 95% CI or p-values given?______

Summary:

1. **Non-random error sufficiently low?** (AMBOM: amount & direction of bias): The study used standard (best possible quality) methodology of population studies. For outcome asthma evaluation data were self-reported, but using ISAAC. For the PA questionnaire, validation or reliability was not noted
2. **Analytical error sufficiently low?** (AN: ITT /adjusted analyses): Possibly, analyses adjusted for: Age, BMI, dietary variables, family asthma or rhinitis, mold in child's bedroom, parental education and smoking, person filling questionnaire, regular sports, season, gender, study centre, TV-viewing
3. **Random error sufficiently low?** (95% CIs: and if no statistically significant effects demonstrated was study power/sample size sufficiently high): The study aimed at body mass index associations; therefore we were unable to extract PA exposure data for this appraisal. The study showed positive associations between low PA (1-2 times per week) and current asthma, but no significant association or trend were present (please refer to data from Corbo et al., 2008, Table 4, cited above). The study could have been underpowered to demonstrate PA-asthma associations
4. **Size of effects sufficient to be meaningful?** (RR &/or RD): N/A
5. **If 1-4 ok, are findings applicable in practice?** (R): Though impairment in assessments for the current appraisal existed, these population data contributed data for time point comparisons (SIDRIA phase I and II) and may be of importance to policy makers, future research (hypothesis generation), and clinical context

GATE Calculator - Risk Factor Cohort Studies

Step 3: Appraise study using PECOT framework (fill in this Calculator in conjunction with appropriate GATE CAT)

a. "hang" the study numbers on the GATE (Graphic Appraisal Tool for Epidemiology) Frame

Assessed by:	LL	Assessed when:	14-Nov-14	Publication details:	**Kosti et al., 2012**

Notes for use:

Enter study numbers in yellow areas.
Help notes appear in moveable boxes.

Enter study descriptions in pink areas

The form calculates results and displays them in the green areas below.

If performing multiple analyses (e.g. reporting on more than one exposure) select tabs 'Analysis 2', 'Analysis 3', etc from the bottom left of the screen

Use together with page 2 of the GATE CAT Risk Factor Cohort Studies form

Populations

Study Setting

Eligible population

Participant population: 1125

Participant subgroup: Urban 700, rural 425

Exposure & Comparison

	Exposure factor (EG)	Comparison factor (CG)
	**Physical activity leisure**	No leisure physical activity
**Numbers allocated to EG & CG:**		
**Follow-up:**		
completed follow-up:	649	476
drop-outs / lost during follow-up:		
**Percentage lost to follow up:**		

Outcomes

Outcome

If categorical.... what e.g. death? Asthma symptoms

	(EG)	(CG)
participants with outcome:	a	b
without outcome:	c	d

If numerical.... what e.g. BP? **Outcome**

	(EG)	(CG)
mean:		
standard deviation:		
or, standard error:		

Report results per (e.g. per 100): 100 persons

Calculated in GATE frame

Results (unadjusted) with 95 % confidence intervals Z-score: 1.96

	Occurrence per 100 persons in exposure group (EGO)	Occurrence per 100 persons in comparison group (CGO)	Exposure effects per 100 persons: Relative effect (EGO/CGO)	Exposure effects per 100 persons: Absolute effect (EGO-CGO)	Number needed to expose (NNE) to prevent/cause 1 event
Categorical outcome: ntention to follow-up analyses 95% CIs					
Categorical outcome: Completed f/u analyses 95% CIs					
Numerical outcome: Analysis of means 95% CIs					

Please contribute your comments and suggestions on this form to: rt.jackson@auckland.ac.nz

Downloadable from www.epiq.co.nz
Copyright © 2004 Rod Jackson, University of Auckland

GATE-lite for RCTs & Observational (risk, prognosis, x-sectional) Studies 2012 and 2013

Study details: Kosti et al., 2012

STUDY QUESTION & DESIGN: describe with PECOT	STUDY NUMBERS: hang on GATE frame	STUDY ERROR: assess using RAMBOMAN
**P = Participants**:	Setting	**Recruitment** appropriate to study goals?
Describe:<br>- Setting: Children from 18 urban and 10 rural schools, context: PANACEA* study. Cross-sectional, Europe (Greece)<br>*Physical activity, Nutrition and Allergies in Children examined in Athens<br>- Eligibility criteria: Age range 10-12 years. Premenstrual girls only<br>- Recruitment process: Schools randomly selected (from list of regional education offices)<br>- % of eligibles who participated: Overall participation rate 83.5%	Eligibles<br>n =_______<br>P<br>n = 700 Urban<br>425 Rural	Setting/eligible population appropriate, given study goals? Yes<br>Participants representative of Eligibles? -<br>Participant risk/prognostic profile reported? Yes
**EG = Exposed** Group [Intervention/Risk factor]	Allocated: randomly or by measurement	**Allocation** (± adjustment) to EG & CG successful/done accurately?
Method of allocation<br>Describe E (how measured if not RCT):<br>Physical activity (PA) "yes" from questionnaire on: Leisure-time activities (e.g., outdoor: walking, cycling; team sports: soccer, swimming) (times per week, outside school hours)	EG Allocated =_____ CG Allocated =_____<br>EG CG	If allocated randomly: Was process concealed? Were EG&CG similar? -<br>If allocated by measurement: Was it done accurately? Done before outcomes? Were differences between EG&CG documented? N/A
**CG = Comparison** Group [Control/comparison]	EG completed follow-up (f/u) =_649__ CG completed f/u =_476__	**Maintenance** of EG & CG as allocated sufficient?
Describe C (how measured if not RCT)<br>No leisure-time PA		Compliance high, Contamination low? Yes, N/A<br>Co-interventions similar in EG&CG? N/A<br>Completeness of follow-up high? Yes<br>Participants/Investigators blind to Exp. status? No
**O =Outcomes:** Primary (& 2° include adverse)<br>**T = Time** when outcomes counted (at what point in time or over what time period)	EG incomplete f/u =_____ CG incomplete f/u =____	**Blind** and **Objective Measurements?**
Describe O & T: how / when measured<br>O. Asthma symptoms of ISAAC (International Study of Asthma and Allergies in Childhood) questionnaire. E.g.: Ever asthma, ever wheeze<br>T: Study took place 2005-2009, sampling equally distributed between Sep and May for all areas	+ a b<br>Could not derive numbers<br>- c d	Outcomes measured accurately? Yes

STUDY ANALYSES	Outcome & Time	EGO = a/EG	CGO = b/CG	RR = EGO/CGO ± 95% CI	RD = EGO-CGO ± 95% CI	NNT = 1/RD ± 95% CI
	Kosti et al., 2012, Table 3. Association [OR (95%CI)] between leisure-time activities (yes/no) and asthma symptoms: 0.90 (0.79, 1.03) (Ns)	-				

ANalyses: Intention to treat (if RCT)?_______ Adjusted if EG & CG different?_______ 95% CI or p-values given?_______

Summary:

1. **Non-random error sufficiently low?** (AMBOM: amount & direction of bias): This is indeed possible. The study used a special validated questionnaire (article reference no. 19: Argiropoulou et al. J Sports Sci Med 2004; 3: 147-159) to record physical activities and the questions were read by the researcher to each participating child
2. **Analytical error sufficiently low?** (AN: ITT /adjusted analyses): Possibly ok, analyses for article's Table 3 adjusted for: Age, gender, body mass index, diet index (KIDMORE)# (Model 3); covariables of Model 3 plus urban/rural factor (Model 4)
#KIDMORE: Mediterranean Diet Quality Index for children and adolescents (total scores and categories described in article)
3. **Random error sufficiently low?** (95% CIs: and if no statistically significant effects demonstrated was study power/sample size sufficiently high): Possibly ok, referral to power calculation ~80% noted by the article Authors because sample size was relatively small
4. **Size of effects sufficient to be meaningful?** (RR &/or RD): N/A
5. **If 1-4 ok, are findings applicable in practice?** (R): The Gate assessment was impaired as data required for this appraisal were not complete; but these results may still be important i.e. applicable to future research (hypothesis generation), clinical practice, and if confirmed by more investigations to policy making

GATE Calculator - Risk Factor Cohort Studies

Step 3: Appraise study using PECOT framework (fill in this Calculator in conjunction with appropriate GATE CAT)

a. "hang" the study numbers on the GATE (Graphic Appraisal Tool for Epidemiology) Frame

Assessed by:	LL	Assessed when:	17-Nov-14	Publication details:	**Mitchell et al., 2013 (data 6-7 plus 13-14 year-old children)**

Notes for use:

Enter study numbers in yellow areas. Help notes appear in moveable boxes.

Enter study descriptions in pink areas

The form calculates results and displays them in the green areas below.

If performing multiple analyses (e.g. reporting on more than one exposure) select tabs 'Analysis 2', 'Analysis 3', etc from the bottom left of the screen

Use together with page 2 of the GATE CAT Risk Factor Cohort Studies form

Populations

Study Setting

Eligible population

Participant population 277534

Participant subgroup

Exposure & Comparison

	Exposure factor (EG)	Comparison factor (CG)
	**Physical activity 1-2x/wk**	Vig PA never/occational /wk
**Numbers allocated to EG & CG:**		
**Follow-up:**		
completed follow-up:	28523	10571
drop-outs / lost during follow-up:		
**Percentage lost to follow up:**		

Outcomes

		EG	CG
**If categorical....** what e.g. death?	**Outcome**: Asthma ever		
participants with outcome:		a	b
without outcome:		c	d
**If numerical....** what e.g. BP?	**Outcome**		
mean:			
standard deviation:			
or, standard error:			

Report results per (e.g. per 100): 100 persons

Results (unadjusted) with 95 % confidence intervals

Z-score: 1.96

Calculated in GATE frame

	Occurrence per 100 persons in exposure group (EGO)	Occurrence per 100 persons in comparison group (CGO)	Exposure effects per 100 persons: Relative effect (EGO/CGO)	Exposure effects per 100 persons: Absolute effect (EGO-CGO)	Number needed to expose (NNE) to prevent/cause 1 event
Categorical outcome: ntention to follow-up analyses					
95% CIs					
Categorical outcome: Completed f/u analyses					
95% CIs					
Numerical outcome: Analysis of means					
95% CIs					

Please contribute your comments and suggestions on this form to: rt.jackson@auckland.ac.nz

Downloadable from www.epiq.co.nz
Copyright © 2004 Rod Jackson, University of Auckland

GATE-lite for RCTs & Observational (risk, prognosis, x-sectional) Studies 2012 and 2013

Study details: Mitchell et al., 2013

STUDY QUESTION & DESIGN: describe with PECOT	STUDY NUMBERS: hang on GATE frame	STUDY ERROR: assess using RAMBOMAN
**P = Participants**:	Setting	**Recruitment** appropriate to study goals?
Describe:<br>- Setting: ISAAC (International Study of Asthma and Allergies in Childhood) Phase Three: Multi-centre, multi-country, cross-sectional study (6-7 year-olds: 29 centres, 17 countries) (13-14 year-olds: 73 centres, 35 countries)<br>- Eligibility criteria: Children at age 6-7 and 13-14 years<br>- Recruitment process: Random sample of schools in defined geographical area<br>- % of eligibles who participated:<br>Age 6-7 years: 34.6%<br>Age 13-14 years: 55.7%	Eligibles<br>n = 220407 (6-7 years)<br>361599 (13-14 years)<br>P<br>n = 76164 (6-7 years)<br>201370 (13-14 years)	Setting/eligible population appropriate, given study goals? Yes<br>Participants representative of Eligibles? Yes<br>Participant risk/prognostic profile reported? Yes
**EG = Exposed** Group [Intervention/Risk factor]	Allocated: randomly or by measurement	**Allocation** (± adjustment) to EG & CG successful/done accurately?
Method of allocation<br>Describe E (how measured if not RCT):<br>Physical activity (PA): 1-2 times per week of vigorous activity long enough to make child breathe hard [using ISAAC (International Study of Asthma and Allergies in Childhood) questionnaire]<br>For 6-7 year-olds: Questionnaire completed by parents or guardians (28% children had objectively measured height and weight)<br>For 13-14 year-olds: Questionnaire was self-completed including reports on height and weight<br>(24% adolescents had objectively measured height and weight)	EG Allocated =_____ CG Allocated =_____<br>EG / CG<br>EG completed follow-up (f/u) / CG completed f/u<br>(6-7y) 8531(39.8%) / 3880(18.1%)<br>(13-14y)19992(49.0%) / 6691(16.4%)	If allocated randomly: Was process concealed? Were EG&CG similar? -<br>If allocated by measurement: Was it done accurately? Done before outcomes? Were differences between EG&CG documented? N/A
**CG = Comparison** Group [Control/comparison]		**Maintenance** of EG & CG as allocated sufficient?
Describe C (how measured if not RCT)<br>Vigorous PA never or occasionally each week	EG incomplete f/u =_____ CG incomplete f/u =_____<br>+ a / b<br>Data not given in article or available (author contact)<br>- c / d	Compliance high, Contamination low? Yes, N/A<br>Co-interventions similar in EG&CG? N/A<br>Completeness of follow-up high? Yes<br>Participants/Investigators blind to Exp. status? No
**O =Outcomes:** Primary (& 2° include adverse)<br>**T = Time** when outcomes counted (at what point in time or over what time period)		**Blind** and **Objective Measurements?**
Describe O & T: how / when measured<br>O: Ever asthma using ISAAC (International Study of Asthma and Allergies in Childhood) questionnaire<br>T: Data collection times not provided. Manuscript first submitted Jan 2012		Outcomes measured accurately? Yes

STUDY ANALYSES

Outcome & Time	EGO = a/EG	CGO = b/CG	RR = EGO/CGO ± 95% CI	RD = EGO-CGO ± 95% CI	NNT = 1/RD ± 95% CI
Mitchell et al., 2013, Table 3: Associations [OR (95%CI)] between reported asthma ever and PA once or twice per week versus vigorous PA never or occasionally each week (reference group):<br>0.96 (0.89, 1.04) (6-7 years)<br>1.14 (1.08, 1.20) (13-14 years)	-				

ANalyses: Intention to treat (if RCT)?______ Adjusted if EG & CG different?______ 95% CI or p-values given?______

Summary:

1. **Non-random error sufficiently low?** (AMBOM: amount & direction of bias): Could be, but inherent limitations from self-reports (asthma, PA)
2. **Analytical error sufficiently low?** (AN: ITT /adjusted analyses): Possibly ok, analyses adjusted for: Body mass index, income, language, region, gender, TV-viewing
3. **Random error sufficiently low?** (95% CIs: and if no statistically significant effects demonstrated was study power/sample size sufficiently high): The results presented in article's Table 3 are likely for both age groups, for example, in the 13-14 year-old group there was some positive association but no dose-response relationship between PA (1-2 and 3-4 times per week versus none) and asthma ever; also credible that there were variations from results for children
4. **Size of effects sufficient to be meaningful?** (RR &/or RD): N/A
5. **If 1-4 ok, are findings applicable in practice?** (R): The Gate assessment of this study was impaired as some data required for calculator were not available, but the cross-national nature of the study design (possibly best current representativeness available for such research data) make the study results important. That is, applicable to future research (hypothesis generating), country or regional specific clinical practice, and health policies of regions
